# A cytoskeleton regulator AVIL drives tumorigenesis in glioblastoma

**DOI:** 10.1038/s41467-020-17279-1

**Published:** 2020-07-10

**Authors:** Zhongqiu Xie, Pawel Ł. Janczyk, Ying Zhang, Aiqun Liu, Xinrui Shi, Sandeep Singh, Loryn Facemire, Kristopher Kubow, Zi Li, Yuemeng Jia, Dorothy Schafer, James W. Mandell, Roger Abounader, Hui Li

**Affiliations:** 1 Department of Pathology, School of Medicine, University of Virginia, Charlottesville, VA 22908 USA; 2 Department of Biochemistry and Molecular Genetics, School of Medicine, University of Virginia, Charlottesville, VA 22908 USA; 3 Department of Microbiology, Immunology, and Cancer Biology, School of Medicine, University of Virginia, Charlottesville, VA 22908 USA; 4 Tumor Hospital, Guangxi Medical University, Nanning, 530021 China; 5 Department of Biology, James Madison University, Harrisonburg, VA 22807 USA; 6 Department of Orthopedics, The Second Xiangya Hospital, Central South University, Changsha, 410011 China; 7 Department of Biology, University of Virginia, Charlottesville, VA 22908 USA

**Keywords:** Cancer genetics, Cancer genetics, Oncogenes, Oncogenes

## Abstract

Glioblastoma is a deadly cancer, with no effective therapies. Better understanding and identification of selective targets are urgently needed. We found that advillin (AVIL) is overexpressed in all the glioblastomas we tested including glioblastoma stem/initiating cells, but hardly detectable in non-neoplastic astrocytes, neural stem cells or normal brain. Glioma patients with increased AVIL expression have a worse prognosis. Silencing AVIL nearly eradicated glioblastoma cells in culture, and dramatically inhibited in vivo xenografts in mice, but had no effect on normal control cells. Conversely, overexpressing AVIL promoted cell proliferation and migration, enabled fibroblasts to escape contact inhibition, and transformed immortalized astrocytes, supporting AVIL being a bona fide oncogene. We provide evidence that the tumorigenic effect of AVIL is partly mediated by FOXM1, which regulates LIN28B, whose expression also correlates with clinical prognosis. AVIL regulates the cytoskeleton through modulating F-actin, while mutants disrupting F-actin binding are defective in its tumorigenic capabilities.

## Abstract (web summary)

Genes that modulate the cytoskeleton have been associated with increased cell proliferation and migration. Here, the authors show that AVIL, an actin regulatory protein, is overexpressed in glioblastomas and mediates oncogenic effects through regulation of FOXM1 stability and LIN28B expression.

## Introduction

Glioblastoma (GBM) is the most common primary brain tumor and among the deadliest of human cancers. Despite advances in surgery, radiation and chemotherapy, survival of patients affected by GBM remains dismal (~15 months after diagnosis)^1–3^. Clearly, better treatment options, and identification of selective therapeutic targets are urgently needed.

Oncogene addiction describes a phenomenon according to which tumor cells become reliant on the activity of a particular oncogene and die once this activity is inhibited^4,5^. Many of the targeted cancer therapies exploit this concept^6^. It is perhaps best exemplified by the successful use of imatinib in the therapy of chronic myelogenous leukemia (CML)^7^. In CML, the major driver of tumorigenesis is the *BCR-ABL* fusion oncogene; imatinib inhibits the constitutively active BCR-ABL protein kinase, to which leukemic cells become addicted. Other successful examples include trastuzumab targeting *ERBB2* addiction^8^, and vemurafenib targeting BRAF addiction^9^. The challenge is to find such key oncogenes. Even though large sets of genome and transcriptome data are available to facilitate the identification of driver mutations in cancer, true signals are often buried in a large number of passenger events. In contrast to adult cancers, pediatric tumors tend to have fewer point mutations and structural changes. While studying a pediatric cancer, rhabdomyosarcoma, we discovered a gene fusion, which results in the juxtaposition of a house-keeping gene next to the *AVIL* gene. Suspecting that other tumors may also dysregulate AVIL expression, we examined AVIL in adult cancers and found its critical role in the tumorigenesis of GBM. We believe that the same approach can be applied to the discovery of other oncogenes.

The cytoskeleton of the cells plays important roles in addition to maintain the cell size and shape. Many critical processes including cell proliferation, migration, and even transcriptional regulations have been connected to the cytoskeleton^10^. Various genes that modulate cytoskeleton have been associated with enhanced infiltrative and proliferative capacity^11^. For instance, in GBM, CTTN, an actin nucleating factor is overexpressed, and this overexpression is associated with an enhanced infiltrative capacity, and poor prognosis^12,13^. Here, we report an oncogene, AVIL, which encodes a protein that regulates F-actin dynamics and cytoskeleton. We found that AVIL is overexpressed in GBM cells including GBM stem cells, and that AVIL overexpression is crucial for GBM proliferation and migration. Mechanistically, AVIL functions upstream of FOXM1. FOXM1 is a member of FOX family. While it is silenced in differentiated cells, it is overexpressed in a number of solid tumors including GBMs^14^. It has been also reported to mediated critical processes of tumorigenesis, such as tumor invasion, angiogenesis, and metastasis^14–18^. On the other hand, let-7 family of microRNAs functions as tumor suppressors and inhibits glioma malignancy^19^. We showed multiple lines of evidence supporting that AVIL regulates FOXM1 stability, which in turn regulates LIN28B/let-7. These findings support the critical role of cytoskeleton dynamics in GBMs, and connect cytoskeleton regulation to the stability of FOXM1 and let-7 expression.

## Results

### AVIL is frequently upregulated in glioblastomas

**Fig. 1:**
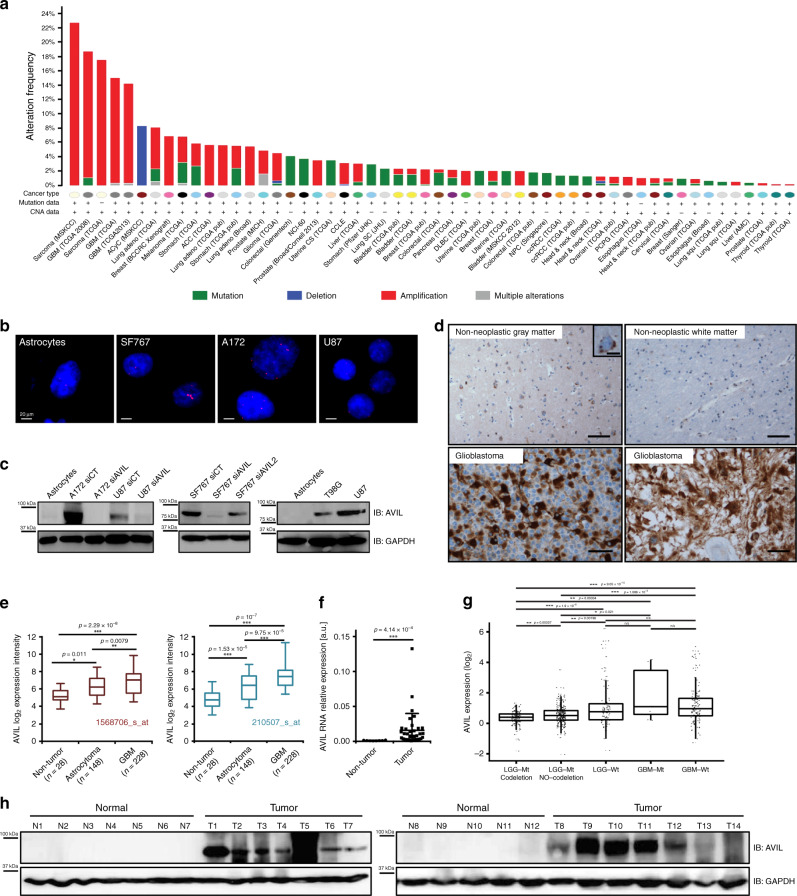
AVIL is overexpressed in glioblastomas. **a** Cross-cancer analysis of mutations and copy number variation from cBioPortal. The *AVIL* locus is amplified in about 15% (TCGA GBM provisional), or 18% (TCGA GBM 2008 study) of GBMs. **b** FISH analysis using a probe covering the *AVIL* locus in GBM cell lines SF767, A172, U87, and an immortalized astrocyte culture. **c** Western blot measuring *AVIL* protein expression in GBM cells, astrocytes, siAVIL treated GBM cells. GAPDH was used as an internal loading control. **d** Immunohistochemical staining of AVIL in non-neoplastic brain and glioblastomas. Scale bars in A-D represent 50 microns; scale bar in the insert represents 10 microns. **e** *AVIL* expression in REMBRANDT database with microarray data of 28 non-tumor brain tissues, 148 astrocytomas (WHO grade II or III), and 228 GBM (WHO grade IV) cases. Results from two microarray probes are shown. **f** qRT-PCR summary of our own collection consisted of 8 non-tumor brain tissues, and 36 GBM cases. *AVIL* level was normalized against that of *GAPDH*. Data are presented as mean values ± SD. **g** RNA-Seq analysis on diffuse glioma study indicates that GBMs have higher level of AVIL than LGG in general, but LGGs with wild-type IDH1 have a comparable high level of AVIL to GBMs. LGG with IDH MT without 1p/19q codeletion is slightly higher than the group with codeletion. **h** Western blot measuring AVIL protein in 12 non-tumor brain biopsies from epilepsy patients (N1-N12), and 14 GBM (T1-T14) samples. For box plots in **e** and **g**, box, 25–75 percentile; lower whisker, least data value excluding outliers; upper whisker, highest data value excluding outliers; bar in middle, median. Lower outliers are calculated as less than Q1 − 1.5*IQR; Upper outliers are calculated as greater than Q3 + 1.5*IQR, where Q1 is value corresponding to 25th percentile and Q3 is value corresponding to 75th percentile and IQR is interquartile range. For all qPCR, *P* value was calculated by standard two-tailed *t*-test. *p < 0.05, **p < 0.01, ***p < 0.001.

Previously, we identified a gene fusion in alveolar rhabdomyosarcoma, a pediatric cancer^20^. We noticed that even though *PAX3-FOXO1* is the most well-known fusion in this type of rhabdomyosarcoma, *MARS-AVIL* has the highest number of reads in the RNA-Seq data (Supplementary Fig. 1a). *MARS* encodes methionyl-tRNAsynthetase. It is a house-keeping gene, expressed in all examined tissues (Supplementary Fig. 1b). AVIL is known as a member of the villin/gelsolin family, that regulates actin filament reorganization^21^. The expression of *AVIL* is more restricted, being low or undetectable in most tissues (Supplementary Fig. 1c). As with many gene fusions, including *IGH-MYC*, and *TMPRSS2-ERG*, the expression of a proto-oncogene may be misregulated by subjecting itself to the control elements of its fusion partner. We suspected that fusion to *MARS* in rhabdomyosarcoma is one mechanism to misregulate *AVIL* gene expression, and that *AVIL* may be misregulated by other mechanisms in other cancers. We found that the *AVIL* locus is amplified in 15–18% of glioblastoma cases in The Cancer Genome Atlas (TCGA) studies^22,23^ via cBioPortal analysis (Fig. 1a). We confirmed such a copy number gain by FISH analyses, using a probe covering the *AVIL* locus (Fig. 1b). The *AVIL* locus is amplified in two glioblastoma cell lines, SF767 and A172, but not in three other glioblastoma lines, U87, U251, T98G, or in an immortalized astrocyte culture.

12q13-15 is frequently amplified in multiple tumor types including glioma^24^. *AVIL* is located on 12q14, 45 kb away from *CDK4* toward centromere, and around 10 MB from *MDM2* toward telomere. Given the importance of *CDK4* and *MDM2* in tumorigenesis, it is possible that *AVIL* amplification is only a byproduct of a larger fragment amplification. However, AVIL expression seems to be upregulated through additional mechanisms: at the protein level AVIL was not detected in the normal astrocytes; in contrast, all of the tumor cell lines tested had higher AVIL protein expression, including the ones that do not have *AVIL* locus amplification (Fig. 1c). This difference was not obvious at the RNA level (Supplementary Fig. 2), suggesting that some post-transcriptional mechanism may be at play.

The overexpression of AVIL protein in GBMs was further confirmed by immunohistochemistry assays. Staining in normal human cerebral cortical gray matter was low, and largely restricted to a subpopulation of pyramidal neurons. Normal white matter demonstrated minimal immunoreactivity. In contrast, all the glioblastomas we tested had much stronger signals (Fig. 1d, and Supplementary Fig. 3).

We then examined the REMBRANDT database, which has microarray data for 28 non-tumor brain tissues, 148 astrocytomas (WHO grade II or III), and 228 GBM (WHO grade IV) cases^25^. Two different microarray probes showed that *AVIL* expression correlates with tumor grade, with the highest levels in GBMs (Fig. 1e). In our own collection of eight non-tumor brain tissues, and 36 glioblastoma cases, we also confirmed the significant difference in *AVIL* RNA expression levels between the two groups (Fig. 1f). To examine the expression levels of AVIL across the major subclasses of glioma recently established and implemented in diagnostic neuropathology, we interrogate the RNA-sequencing dataset from the TCGA diffuse glioma study^26^. Here we subdivided the TCGA samples into GBMs with IDH-wild type, GBMs with IDH-mutant, Lower-grade glioma (LGG) with IDH-wild type, LGG with mutant IDH and 1p/19q codeletion, and LGG with IDH mutant without 1p/19q codeletion. Consistent with REMBRANT data, GBMs with or without IDH mutation have high level of AVIL expression. Within LGG, the group with IDH WT is the highest and not statically different from the GBMs. LGG IDH MT without 1p/19q codeletion is slightly higher than the group with codeletion (Fig. 1g).

We then examined the AVIL protein in 12 non-tumor brain tissues, and 14 GBMs. The AVIL protein was absent, or barely detected in the non-tumor cases, but higher levels of AVIL protein expression were seen in almost all of the GBM cases (Fig. 1h). These results further suggest that in addition to the 15–18% of cases that have copy number gain, a higher percentage of glioblastomas may use post-transcriptional/translational mechanisms to up-regulate AVIL expression. They provide indirect evidence that AVIL overexpression may be involved in GBM tumorigenesis rather than being solely a passenger event, a consequence of *CDK4* and *MDM2* locus amplification.

In order to determine whether AVIL protein expression levels correlate with tumor grade and/or IDH mutation status, we performed a semiquantitative analysis of AVIL IHC data on a set of diffuse gliomas, consisting of grade II astrocytomas and oligodendrogliomas (*n* = 11, grade III astrocytomas and oligodendrogliomas (*n* = 14) and grade IV glioblastomas (*n* = 15) (Supplementary Fig. 3i). Using a 0–3 scoring system (1 => 0% <10% tumor cells positive; 2 => 10% <50% tumor cells positive; 3 => 50% tumor cells positive), we found a positive correlation with grade. WHO grade II tumors mean = 1.14 ± 0.40 (SEM); WHO grade III tumors mean = 1.93 ± 0.23 and WHO Grade IV tumors (glioblastoma) mean = 2.72 ± 0.18. Using unpaired T-tests, a significant difference is detected between grade IV and each other group (comparison of grade IV and grade III, *p* = 0.0126; comparison of grade IV and grade II, *p* = 0.0072). Because IDH1 mutation status has proven to be a more important prognostic factor than histologic grade, we then compared AVIL IHC scores between IDH1 (R132) mutant and wild-type tumors, with the observer blinded to IDH1 status. We found that IDH1 (R132H) mutant gliomas had a mean score of 1.31 ± 0.31 (SEM) while IDH1 wild-type gliomas had a mean score of 2.36 ± 0.21 (SEM), significantly differing with a two-tailed *P* value of 0.0063 (Supplementary Fig. 3j).

**Fig. 2:**
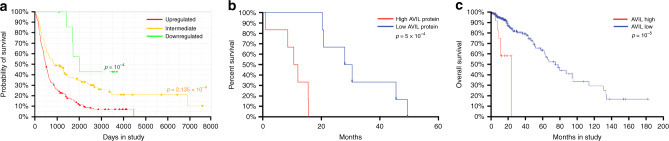
High AVIL expression is correlated with worse clinical prognosis of glioma patients. **a** Clinical analysis using the REMBRANDT dataset. A three-class model, stratified by *AVIL* RNA expression in 343 glioma cases. Higher expression of *AVIL* correlates with worse overall patient survival (*p* = 1 × 10^−4^, two-sided log-rank test). **b** A two-class model stratified by AVIL protein expression in the 14 GBM samples. The high AVIL group has worse overall survival than the low AVIL group (*p* = 5 × 10^−4^, two-sided log-rank test). **c** Clinical analysis using TCGA lower-grade glioma dataset. A two-class model stratified by *AVIL* RNA expression in 286 samples that have RNA-sequencing data. The high *AVIL* group has a much shorter overall survival than the low *AVIL* group (*p* = 1 × 10^−5^, two-sided log-rank test). The median survival for the high *AVIL* group is 23.1 months, versus 75.1 months for the low *AVIL* group.

To further validate the clinical significance of *AVIL* in human gliomas, we examined the relationship between *AVIL* expression, and patient survival in 343 glioma cases in the REMBRANDT project^25^. A three-class model, in which patients were stratified according to *AVIL* expression showed a clear positive correlation between elevated *AVIL* expression, and shorter survival (upregulated vs. intermediate, *p* = 1 × 10^−5^; upregulated vs. all other, *p* = 4 × 10^−7^, log-rank test) (Fig. 2a. The same trend was also observed with a different microarray probe using a two-class model (Supplementary Fig. 4a). Suspecting that this correlation is due to the overall higher expression of *AVIL* RNA in GBM as a group (Fig. 1e), we then focused on GBM cases. Within GBMs in TCGA database, we found no association between *AVIL* RNA level and patient survival (Supplementary Fig. 4b). However, when we examined AVIL protein expression using our collections of clinical GBM collections, we saw a strong inverse correlation between AVIL protein expression and patient survival (*R* = −0.82, *p* = 0.0012) (Supplementary Fig. 4c). Kaplan-Meier survival analyses also demonstrated a correlation between poor prognosis and the high AVIL protein expression (log-rank test *p* = 0.0005) (Fig. 2b).

The term Lower-grade glioma (LGG) includes the grade II and III gliomas. However, the management of LGG is one of the most controversial areas in clinical neuro-oncology, with survival ranges from 1 to 15 years. We queried a recent TCGA lower-grade glioma dataset^27^ for *AVIL* expression. Out of 286 samples that had RNA-sequencing data, the high *AVIL* group (two fold or higher than average) had a much shorter overall survival than the low *AVIL* group (*p* = 1 × 10^−5^, log-rank test). The median survival for the low *AVIL* group was 75.1 months. In contrast, the high *AVIL* group had a median survival of only 23.1 months, comparable to those of GBM patients (Fig. 2c and Supplementary Fig. 5a). Consistently, the two groups also had a significant difference in disease-free survival (*p* < 0.01) (Supplementary Fig. 5b). The difference in patient survival based on *AVIL* expression is more significant than that based on traditional histopathologic classification and grading.

### AVIL overexpression is crucial for GBM tumorigenesis

**Fig. 3:**
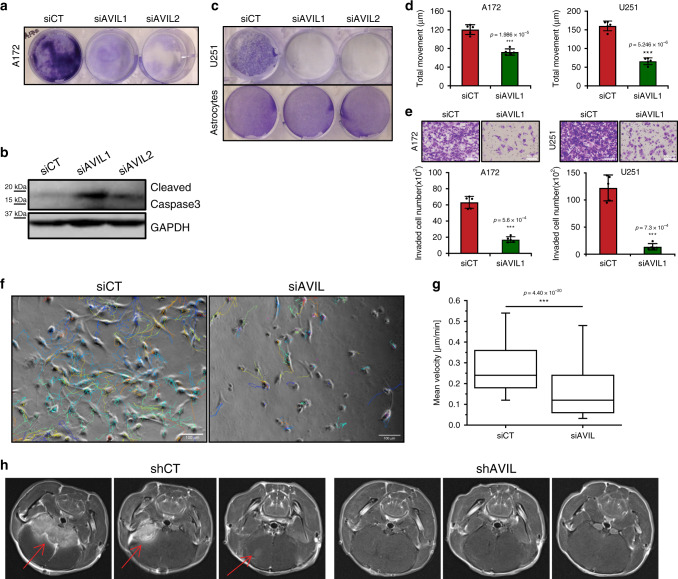
AVIL overexpression is crucial for GBM cell line tumorigenesis. **a** Crystal violet staining of A172 cells transfected with siAVIL1, siAVIL2, or siCT after a week of transfection. **b** Western blot measuring cleaved Caspase3 in A172 cells transfected with the siRNAs. **c** Effect of AVIL silencing in U251 cells (upper) and astrocytes (lower). **d** Wound-healing measuring cell migration at 48 h after siRNA transfection in A172 (left), and U251 cells (right) (*n* = 5, two-sided Student’s *t-*test). **e** Tumor cell invasiveness was measured by matrigel-coated transwell assay at 48 h after transfection. Shown are results from A172 (left), and U251 cells (right) (*n* = 5, two-sided Student’s *t-*test). **f** Movement of A172 cells was observed over 24 h period of time by live-cell imaging, starting 24 h after transection with either siCT (left) or siAVIL (right). Shown are representative images depicting the last timepoint of the experiment with overlaid lines tracking the movement of individual cells. **g** Mean velocities of all cells tracked in the experiment depicted in **f** (*n* > 150 cells quantified per condition) (box, 25–75 percentile; whisker, 5–95 percentile; bar in middle, median) (two-sided Student’s *t-*test). **h** Three representative MRI brain images of mice injected with U251 cells stably expressing shCT or shAVIL. Red arrows point to the area of tumor. Data are presented as mean values ± SD in **d** and **e**. *p < 0.05, **p < 0.01, ***p < 0.001.

We then directly tested whether overexpression of AVIL is necessary for the tumorigenesis of GBM. Two independent siRNAs effectively silenced *AVIL* expression in A172 GBM cells (Supplementary Fig. 6a). By a week, the cells transfected with either AVIL-targeting siRNA almost completely died out (Fig. 3a). Consistently, we observed a significant induction of cleaved Caspase3 around 96–120 h after transfection in these cells (Fig. 3b). The same effect was seen in U251 (Fig. 3c and Supplementary Fig. 6b). In contrast, no growth inhibition was seen in the astrocyte cultures (Fig. 3c and Supplementary Fig. 6c). We also measured the effect of silencing AVIL on the migratory ability and invasiveness of A172 and U251 cells. When AVIL was silenced at 48–72 h, a dramatic reduction in the cell movement was observed by a wound-healing assay in both cell lines (Fig. 3d). Similarly, the number of cells that invaded through trans-well was dramatically reduced in both cell lines (Fig. 3e). The wound healing and trans-well assays were performed at early time points after siRNA transfection, and the duration of the experiments were shorter than the doubling time. Nonetheless, the effect of AVIL silencing on cell survival and proliferation complicates the evaluation of cellular motility. To confirm the effect of AVIL on cell migration, we performed live-cell imaging on A172 cells, following the track of movement for individual cells 24 h after transfection. Consistently, obvious reduction was observed when AVIL was silenced (Fig. 3f, g and Supplementary Movie 1).

To confirm whether AVIL plays an important role in tumorigenesis in vivo, we tested the effect of silencing AVIL in tumor initiation with a widely used U251 intracranial xenograft model. In this model, tumor-bearing animals usually die in about one month^28^. We implanted U251 cells that were freshly infected with lentivirus expressing shAVIL1, or shCT in the brains of immune-deficient mice. After 4 weeks, all control mice had reached significant tumor volumes, detected by MRI (Fig. 3h). In contrast, we could hardly observe any tumor formation in the shAVIL1 group by MRI. Consistently, dramatic differences in tumor volumes were observed between the two groups (Supplementary Fig. 7a). All of the shCT group animals died within 35 days, or had to be euthanized due to abnormal behavior caused by tumor burden. All of the shAVIL1 group animals displayed no sign of disease, until the day we terminated the experiment, with the exception of two (we euthanized one shAVIL1 mouse at the same time as a shCT mouse as a control. Another shAVIL1 mouse developed an infection after the MRI imaging, requiring euthanasia) (Supplementary Fig. 7b). These results support the crucial role that the overexpression of *AVIL* plays in tumorigenesis in vivo.

**Fig. 4:**
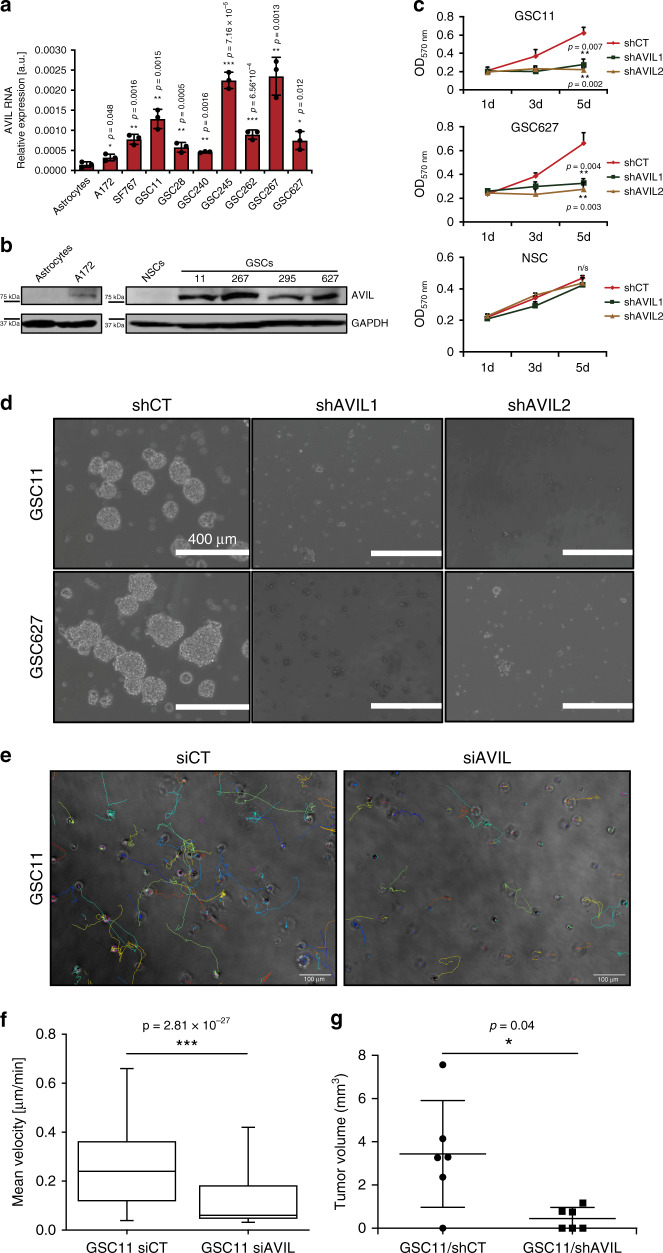
AVIL overexpression is crucial for GSCs/GICs. **a** qRT-PCR measuring *AVIL* mRNA level in GSCs, two GBM cell lines, and control astrocytes. *AVIL* RNA expression was normalized against that of *GAPDH*. **b** Western blot measuring AVIL protein in astrocyte, A172, NSC, and four GSC cell lines. **c** Cell growth measured by MTT. Shown are the results of GSC11 (upper), GSC627 (middle), and NSC (lower) cells transfected with shAVIL1, shAVIL2, or control shCT (*n* = 6). **d** Neurosphere formation was inhibited with AVIL silencing. GSC11 (upper) and GSC627 (lower) cells were transfected with shAVIL1, shAVIL2, or control shCT. **e** Movement of GSC11 cells suspended in Matrigel matrix was observed over 24 h period of time by live-cell imaging, starting 6 h after transection with either siCT (left) or siAVIL (right). Shown are representative images depicting the last timepoint of the experiment with overlaid lines tracking the movement of individual cells. **f** Mean velocities of all cells tracked in the experiment depicted in **e**. *n* > 300 cells quantified per condition (box, 25–75 percentile; whisker, 5–95 percentile; bar in middle, median) (two-sided Student’s *t-*test). **g** Tumor volume comparison between GSC11 cells infected with shCT expressing virus and shAVIL virus (*n* = 6). Data are presented as mean values ± SD in **a**, **c**, and **g**. *P* value was calculated by standard two-tailed *t*-test. *p < 0.05, **p < 0.01, ***p < 0.001.

Like other cancer types, GBMs harbor a subpopulation of glioblastoma initiating/stem cells (GICs/GSCs) that govern tumor initiation, maintenance, and recurrence after therapy^29^. GSCs partly mediate resistance to chemo and radiotherapy, and have become vital targets for the reversal of chemo-resistance^30^. All the GIC/GSC cells in our collection express a high level of AVIL, while a neural stem cell culture^31^ had almost no detectable AVIL expression (Fig. 4a, b). Consistently, we found that silencing AVIL in two GSCs, GSC11 and GSC627, resulted in dramatic reduction of cell proliferation, while no obvious difference was observed in the neural stem cell culture (Fig. 4c). Neurosphere formation was also dramatically reduced in the two GSCs (Fig. 4d). Not surprisingly, the expression of stemness markers was reduced, whereas both astrocyte and oligodendrocyte differentiation markers were induced when AVIL was silenced (Supplementary Fig. 8). Using live-cell imaging, we traced the movement of GSC individual cells in 3D culture. Similar to GBM cells, silencing AVIL had a dramatic effect on cell migration in that GSC-11 cells with AVIL silencing hardly moved during a 24 h period (Fig. 4e, f and Supplementary Movie 2). To evaluate AVIL effect on GSCs in vivo, we silenced AVIL in GSC11 cells and performed the intracranial xenografts. Compared with the shCT control group, shAVIL group had significantly smaller tumors (Fig. 4g). In addition, more apoptotic cells were found in the shAVIL group (Supplementary Fig. 9).

### AVIL is a bona fide oncogene

**Fig. 5:**
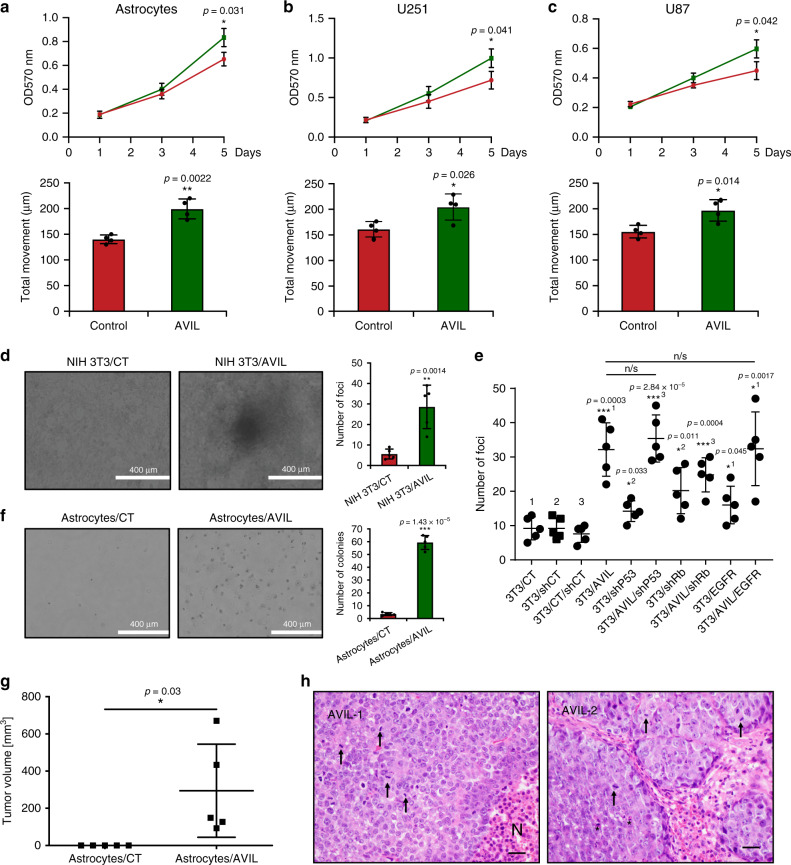
*AVIL* overexpression promotes tumorigenesis. Overexpressing AVIL with Myc-tagged AVIL in astrocytes (**a**), U251 (**b**), and U87 (**c**) resulted in increased cell proliferation, assayed by MTT (upper), or cell motility, assayed by wound-healing (lower). *P* value was calculated by standard two-tailed *t*-test. **d** Focus assay. NIH-3T3 cells were transfected with AVIL expressing (AVIL) or control empty plasmid (CT). Representative image is shown. Two-sided Student’s *t-*test. **e** Quantification of foci resulted from oncogene cooperation assays. NIH-3T3 cells were transfected with shP53, ShRB, EGFR VIII mutant with or without AVIL overexpression. Two-sided Student’s *t-*test. 1, 2, and 3 groups are compared to the corresponding controls. **f** Soft-agar assay showing that astrocytes transfected with AVIL formed a much larger number of colonies compared with those transfected with empty vector control (CT). *P* value was calculated by standard two-tailed t-test. **g** Measurement of the tumor volume. No tumor was formed in the CT group. **h** Representative hematoxylin and eosin staining of the tumors harvested from the mice. Histology analysis revealed neoplasms with histologic features of malignancy including necrosis (N), frequent mitotic figures (arrows), and apoptotic figures (asterisks). *p < 0.05, **p < 0.01, ***p < 0.001. Data are presented as mean values ± SD in **a**–**f**, and **h**. **a**–**c**, *n* = 4; D-G, *n* = 5.

To further confirm that high levels of AVIL are tumorigenic, we examined the effects of AVIL overexpression in astrocyte, U251, and U87 cells. AVIL overexpression led to increased proliferation rates, and increased migration in all three cell types (Fig. 5a–c).

To test whether AVIL can function as a bona fide oncogene, we performed the classic focus assay on NIH3T3 cells expressing, or not, AVIL. We observed significantly larger and higher numbers of foci in cells transfected with AVIL than with an empty vector control (Fig. 5d). Most GBMs have genetic alterations in three core signaling ways^32–34^, including RTK/RAS pathway with alteration in EGFR/PDGFRA/PI3K/PTEN/NF1/RAS, the p53 pathway with genetic alteration in TP53/MDM2/MDM4/p14ARF, and the RB1/CDK4/p16INK4A/CDKN2B pathway. To examine the potential connection and collaborative effect of AVIL with these three pathways, we performed a modified oncogene cooperativity assay, where we introduced alone the EGFR vIII mutant^35^, shRNA targeting TP53, or shRNA targeting RB, or in combination with AVIL overexpression. As shown in Fig. 5e, all three resulted in an increased number of foci. Impressively, overexpressing AVIL alone has triggered a larger number of foci compared with any of these three known oncogenic factors. In addition, combing AVIL with these known factors did not result in more foci, but rather less in the case of shRb and AVIL combination. These results suggest that AVIL is sufficient to trigger focus formation and is at least as powerful, if not stronger, than the known factors.

To evaluate the transforming ability of AVIL in vitro, we performed soft agar assay to test anchorage independency for astrocytes overexpressing AVIL. Compared with empty vector-transfected controls, a large number of colonies were observed in astrocytes transfected with an AVIL expressing vector (Fig. 5f).

Lastly, we tested whether the overexpression of AVIL would transform astrocytes in vivo. We injected 2 million astrocyte cells stably expressing either AVIL or a control plasmid subcutaneously into the flanks of NIH-III nude mice. No tumor was seen in any of the ten injections for the control group. In contrast, five out of 14 injections of the AVIL overexpression group had visible tumors within ten days of injection (5G). To confirm that the masses formed in the group of AVIL overexpression are truly tumor, we examined them histologically. Indeed, the cells from AVIL overexpressed astrocytes revealed a neoplasm with histologic features of malignancy including necrosis (N), frequent mitotic figures (arrows), and apoptotic figures (asterisks) (Fig. 5h). We then conducted RNA-Seq on the whole transcriptome changes, and identified a large number of gene expression affected. Among them, all these major oncogenic pathways involved in GBM tumorigenesis were enriched by a Gene Set Enrichment Analysis (GESA) (Supplementary Fig. 10). On the other hand, we found no significant changes of AVIL expression when we introduced EGFR mutant, shTP53 or shRB into these astrocytes (Supplementary Fig. 11). These results together with our observation that AVIL is overexpressed in all GBMs we tested, suggest that AVIL overexpression is a common theme, and it may even function upstream of the three known oncogenic signaling pathways.

### AVIL binds to F-actin

**Fig. 6:**
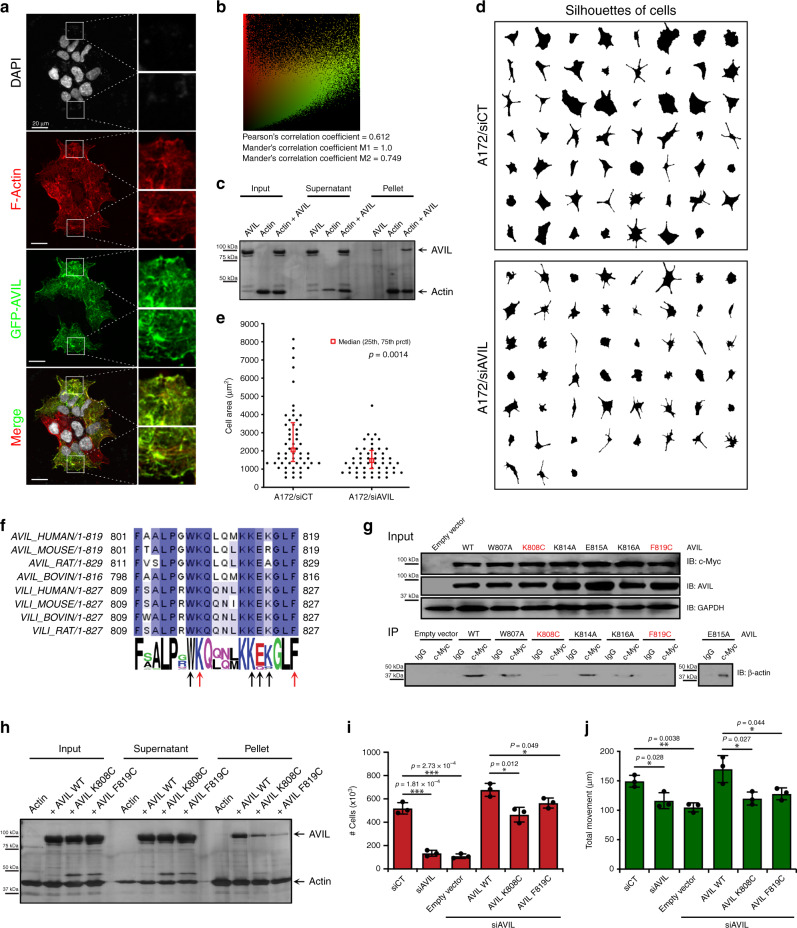
AVIL binds to F-actin, and the interaction is crucial for its tumorigenic activity. **a** AVIL colocalize with F-actin. U87 cells stably expressing GFP-AVIL (green) were stained for F-actin (Phalloidin, red) showing co-localization of AVIL and F-actin. **b** Fluorescence colocalization between F-actin and AVIL. Scatterplot representing the intensity range of red and green pixels in the image as shown in **a**. **c** AVIL binds directly to F-actin. Silver-stained SDS-PAGE analysis of actin high-speed pelleting of F-actin in the presence or absence of AVIL. **d** Silencing AVIL resulted in reduced ability of A172 cells to spread. **e** Cell area was plotted for A172 cells transfected with siCT, or siAVIL1. Significant difference (*p* = 0.0014) was observed by the two-sided Rank-Sum test (non-parametric test for non-normally distributed data). *n* > 50. **f** Amino acid sequence alignment of the C-terminal region of advillin and villin from eight species. Arrows indicate positions selected for mutagenesis; red arrows indicate positions of mutations used in experiments represented in **g**–**j**. **g** Mutations in the headpiece region of AVIL significantly reduce binding to β-actin in cells. Co-immunoprecipitation of β-actin by antibodies specific to Myc-tag in cell lines expressing Myc-tagged AVIL WT or indicated mutants. Mutants indicated in red were subsequently utilized in experiments represented in **h**–**j**. IB – immunoblotting, IP – co-immunoprecipitation. **h** AVIL mutants are deficient in binding to F-actin. Silver-stained SDS-PAGE analysis of actin high-speed pelleting of F-actin in the presence or absence of AVIL in a buffer containing EGTA. **i** Mutations in AVIL headpiece region are not as effective as WT AVIL in stimulating cell proliferation (two-sided Student’s *t-*test). U87 cells were transfected with siAVIL and grown for 24 h followed by transfection with a plasmid overexpressing AVIL WT or indicated AVIL mutants. After 48 h, cells were counted. **j** Headpiece mutants failed to rescue deficiency in cell movement (two-sided Student’s *t-*test). Total movement of U87 cells expressing AVIL WT or indicated mutants in the wound-healing experiment. Data are presented as mean values ± SD in **e**, **i**, and **j**.

AVIL is a member of the villin/gelsolin family of actin-regulatory proteins^21^. The gene encodes a protein also known as advillin, which has been reported to affect cell movement, and is involved in the formation of filopodia-like structures in fibroblasts, as well as a role in ciliogenesis^36^. Consistently, our results showed that silencing AVIL reduced migration in GBM cells, whereas overexpression enhanced their migration (Figs. 3 and 5). However, the exact activities of AVIL on actin cytoskeleton have not been extensively examined. We first visualized colocalization of GFP-tagged AVIL, with phalloidin staining of F-actin (Fig. 6a, b and Supplementary Fig. 12). We confirmed direct binding of recombinant AVIL to actin filaments by high-speed ultracentrifugation (Fig. 6c and Supplementary Fig. 13). More AVIL was found to be in the pellet fraction when actin was added.

### AVIL’s activity depends on F-actin binding

The dependence of GBM cells on AVIL to attach and spread was further evidenced when we monitored the cell shape and area when newly plated onto fibronectin substrate. Control cells spread much more than the siAVIL1 transfected cells, reflected by overall silhouettes of the cells and the areas cells occupied (Fig. 6d, e).

We further analyzed the effect of AVIL knock-down on the dynamics of cellular protrusions and retractions on the cell edge as those processes were previously established to be dependent on actin cytoskeleton dynamics^37^. Although the knock-down of AVIL did not affect the rate of the membrane retraction, the rate of forward protrusion at the cell edge was increased compared to controls (Supplementary Fig. 14). Even though the reduced migration cannot be directly explained by this defect, the result is consistent with an imbalance in the actin cytoskeleton regulation caused by the loss of AVIL. This finding supports a role for AVIL in the regulation of actin dynamics in cells.

AVIL’s headpiece domain is situated at the C-terminus and shares sequence and structure similarity with villin’s headpiece. The villin headpiece domain was previously shown to be crucial for F-actin binding and bundling^38^. Based on the structural models of the villin and advillin headpiece domains^21^, and sequence conservation, we selected six residues in the AVIL headpiece for mutagenesis and biochemical studies (Fig. 6f). We expressed Myc-tagged AVIL, either wild-type or mutants, in glioblastoma cells in which endogenous AVIL was silenced and performed co-immunoprecipitation with anti-Myc antibody to assess the interaction between actin and AVIL mutants. The majority of the AVIL headpiece mutants exhibited reduced binding to actin (Fig. 6g).

We then chose two mutants (AVIL K808C and AVIL F819C) that yielded the lowest amount of actin in the co-immunoprecipitation experiment and are also highly conserved for further studies. Consistently, both recombinant AVIL mutant proteins exhibited reduced binding to filamentous actin (Fig. 6h and Supplementary Fig. 15). Compared to the wild-type AVIL, these mutants do not promote cell proliferation to the same rate (Fig. 6i). More importantly, the mutants failed to rescue cell migration caused by AVIL silencing (Fig. 6j).

### AVIL regulates the stability of FOXM1

**Fig. 7:**
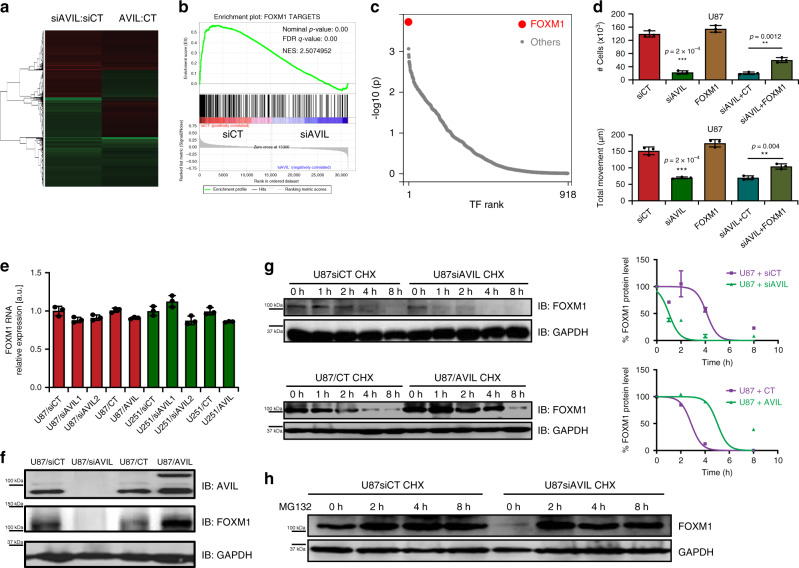
AVIL regulates the stability of FOXM1. **a** Whole transcriptome analyses of U87 cells transfected with siAVIL vs. siCT, and stable AVIL overexpression cells vs. empty vector control (CT). **b** Gene Set Enrichment Analysis revealed highly similar profiles between FOXM1 targets, and differentially expressed genes caused by AVIL silencing and overexpression. NES score = 2.5. **c** BART prediction ranked FOXM1 as the top factor. **d** FOXM1 expression can rescue at least some of the AVIL effect. U87 cells transfected with siAVIL or siCT were further transfected with FOXM1 expression vector, or control plasmid (CT). Cell proliferation was measured by cell counting. Cell motility was measured by wound-healing assay. **e** *FOXM1* RNA expression was not affected by AVIL silencing or overexpression. qRT-PCR measuring *FOXM1* mRNA level in U87 and U251 cells. *FOXM1* RNA expression was normalized against that of *GAPDH*. No statistical significance (ns) was observed in all groups compared to CT or siCT. **f** FOXM1 protein was reduced upon AVIL silencing, and induced upon AVIL overexpression. Western blot measured the protein levels of AVIL, FOXM1, and GAPDH. Upper band in AVIL Western is GFP-tagged AVIL. **g** FOXM1 protein half-life was reduced upon AVIL silencing, and induced upon AVIL overexpression. U87 cells were treated with cycloheximide to inhibit new protein synthesis. FOXM1, and GAPDH proteins were measured by Western Blot analysis. Protein signals were quantified by densitometry. **h** The reduced half-life of FOXM1 can be offset by the proteasome inhibitor, MG132. Data are presented as mean values ± SD in **d**, and **e**.

We then performed whole transcriptome analyses on U87 cells silenced for AVIL, and with AVIL overexpression, together with controls (Fig. 7a). We focused on those candidates whose expression was inversely changed when AVIL was silenced or overexpressed. We noticed that many of such candidates including *AURKA, AURKB, CDC20, KIF11, SPC25*, and *TTK* etc., are known targets of transcription factor, FOXM1. Gene Set Enrichment Analysis (GESA) revealed highly similar profiles between FOXM1 targets^39^, and differentially expressed genes caused by AVIL silencing/overexpression (Fig. 7b). In addition, using BART, a bioinformatics tool for predicting functional factors^40^, FOXM1 was predicted to be the top factor, far more significant than other factors (Fig. 7c). FOXM1 is overexpressed in many cancers including glioma^14^, where it has been demonstrated to play a role in the pathogenesis, progression, and metastasis^15,41^. We found the reduced cell growth and migration abilities caused by AVIL silencing can be at least partially rescued by overexpressing FOXM1 (Fig. 7d), suggesting that FOXM1 functions downstream of AVIL.

Perturbing AVIL had no effect on *FOXM1* mRNA (Fig. 7e), but affected FOXM1 at the protein level (Fig. 7f), suggesting a post-transcriptional mechanism of AVIL on FOXM1. Indeed, FOXM1 half-life was reduced by silencing AVIL, and increased by overexpressing AVIL (Fig. 7g). Given the activity of AVIL on F-actin, we suspect that disturbing F-actin dynamics may also perturb FOXM1 stability. Indeed, when we used F-actin polymerization inhibitor Cytochalasin D, or the depolymerization inhibitor Jasplakinolide to treat U87 cells, we also observed significant reduction of FOXM1, supporting that AVIL loss or wholesale disruption of F-actin dynamic result in reduced FOXM1 protein (Supplementary Fig. 16) Consistently, using proteasome inhibitor MG132, the effect of AVIL silencing on FOXM1 was fully abolished (Fig. 7h).

**Fig. 8:**
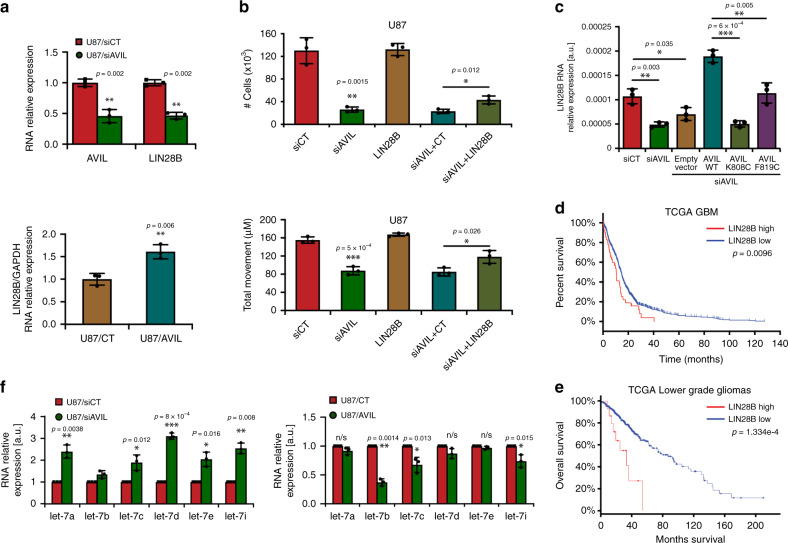
AVIL regulates LIN28B expression through FOXM1. **a** LIN28B was reduced upon AVIL silencing, and induced upon AVIL overexpression. U87 cells were transfected with siAVIL or siCT, and empty vector control (CT) or AVIL expressing vector. *LIN28B* level was measured by qRT-PCR, and normalized to that of *GAPDH*, and then to siCT. **b** LIN28B mediates at least some of the AVIL effect. U87 cells transfected with siAVIL or siCT were further transfected with LIN28B expression vector, or control plasmid (CT). Cell proliferation was measured by cell counting. Cell motility was measured by wound-healing assay. **c** Headpiece mutants are not as effective as WT in inducing *LIN28B* expression. *LIN28B* expression was measured by qRT-PCR and is represented relative to *GAPDH* levels. **d** Clinical correlation between the expression of *LIN28B* and patient survival. A two-class model, stratified by *LIN28B* expression using TCGA GBM data. The *LIN28B* high group has a significantly worse survival (*p* < 0.01, two-sided log-rank test). **e** With TCGA lower-grade glioma data, *LIN28B* high group is found to have worse overall survival (*p* = 0.00013) (two-sided log-rank test). **f** *Let-7* family members were induced upon AVIL silencing, and suppressed upon AVIL overexpression. Let-7 family members that are expressed in U87 were measured by qRT-PCR. Their expression levels were plotted against that of siCT, or CT. *p < 0.05, **p < 0.01, ***p < 0.001. Data are presented as mean values ± SD in **a**–**c**, and **f**.

Among the genes that were inversely regulated by AVIL silencing vs. overexpressing, LIN28B caught our attention. LIN28B belongs to the group of RNA-binding proteins that play critical roles in embryonic development, and in tumorigenesis^42^. Several studies have demonstrated that LIN28B can promote proliferation, and invasion of cancer cells^43,44^. It has been postulated that LIN28B is regulated by FOXM1^26^. Indeed, we found that silencing AVIL or FOXM1 resulted in reduced *LIN28B* expression, whereas overexpressing AVIL or FOXM1 enhanced *LIN28* expression (Fig. 8a and Supplementary Fig. 17). Supporting that LIN28B is downstream of AVIL, we found that LIN28B expression partially rescued both reduced cell proliferation and migration caused by AVIL silencing in U87 and U251 cells (Fig. 8b, and Supplementary Fig. 18). In GSC cells, similar observation was made supporting that LIN28B mediates at least part of tumorigenic effect of AVIL (Supplementary Fig. 19). Consistently, AVIL mutants defective in F-actin binding also failed to induce LIN28B to the same extent as the wild-type AVIL (Fig. 8c). Clinically, high LIN28B expression correlates with poor prognosis in patients with GBMs (Fig. 8d), and lower-grade gliomas (Fig. 8e).

LIN28B is best known to negatively regulate the biogenesis of tumor-suppressive microRNAs, *let-7*. Consistently, let-7 members have been shown to inhibit glioma cell malignancy^19^, and are expressed at lower levels in high-grade gliomas^19^. Indeed, we observed that silencing AVIL induced all of the members of *let-7* that are expressed (*let7-f*, *let7-g*, and *mir-98* are not expressed in U87 cells) (Fig. 8f). Conversely, AVIL overexpression resulted in the down-regulation of most *let-7* members. Recently, the overexpression of several let-7 downstream targets including *HMGA2*, *IGFBP1*, and *IGFBP3*, were found to define a distinct patient group with poor prognosis in pancreatic cancer^45^. HMGA2 and IGFBP1 have been shown to antagonize the tumor-suppressive activity of let-7^46^. Since let-7 expression level is not available in the TCGA datasets, we examined *HMGA2*, *IGFBP1*, and *IGFBP3* level. Impressively, higher levels of all three genes are correlated with worse overall survival, as well as disease-free survival (Supplementary Fig. 20).

## Discussion

Glioblastoma (GBM), WHO classification Grade IV Astrocytoma, is the most common, and most aggressive malignant primary brain tumor in humans^47^. Survival of patients affected by GBM has remained low, despite advances in surgery, radiation, and chemotherapy^1–3^. About 50% of patients diagnosed with GBM die within one year, and 90% die within three years^48^. More therapeutic targets and treatment options are clearly needed. Our study shows that AVIL is overexpressed in the vast majority, if not all of human glioblastomas. We found that GBM cells, including GSC/GIC cells are dependent on the overexpression of AVIL for increased survival and migration. Silencing AVIL induced GBM cell death in vitro, and prevented/reduced GBM xenograft formation and growth in animal models. In contrast, normal astrocytes or neural stem cells express very low levels of AVIL, and silencing AVIL had no obvious effect. Taken together, this demonstrates that AVIL is crucial for GBM tumorigenesis, which are addicted to the AVIL overexpression. Therefore, AVIL may be a promising selective therapeutic target, inhibition of which may effectively suppress GBM growth and invasion, yet spare normal brain cells.

Tumor cells use multiple “tricks” to dysregulate some oncogenes, which at the same time give credence to the genes as key players in tumorigenesis and malignancy. Our study showed that *AVIL* is dysregulated via multiple mechanisms. It forms a fusion with *MARS* in rhabdomyosarcomas. It is amplified by copy number gain in some glioblastomas, and upregulated at the transcriptional/translational level in other GBMs. In this regard, it is similar to anaplastic lymphoma kinase (ALK). *ALK* has been proven to be a remarkably promiscuous oncogene^49^. It contributes to the development of a notable assortment of tumor types from different lineages through a variety of genetic mechanisms. It can form *NPM-ALK* fusions in ~60% of anaplastic large-cell lymphomas (ALCLs)^50^, or *EML-ALK* in a subset of non-small-cell lung cancers^51^; additionally, it can have gain-of-function point mutations in neuroblastomas^52^, and copy number gain in several tumor types^53^. Accordingly, the *ALK* inhibitor, Crizotinib, was proven to be an effective, and safe treatment for lung cancer patients harboring *EML-ALK*^54^, leading to the quick approval of the drug by the FDA. Considering gene fusions often result in aberrant expression of one of the proto-oncogenes, our strategy was to start from a gene fusion in pediatric cancer, then extend to adult tumors including GBM. We believe that the approach may lead to the discovery of other promiscuous oncogenes.

AVIL is known as a member of the villin/gelsolin family, which regulates actin filament reorganization^21^. We demonstrated that F-actin interacting capability of advillin is crucial for the migration of GBM cells, and for its downstream signaling pathways. Advillin is 75% homologous to villin, and 65% homologous to gelsolin and adseverin. Recently, AVIL mutants were found to be involved in the alteration PLCE1 action, and contribute to the Steroid-resistant nephrotic syndrome^55^. Even though there are studies suggesting connections between these actin-regulatory proteins and cancer^56,57^, no direct evidence has shown any of the family members to be oncogenic, or tumor suppressive. However, given the importance of actin dynamic in cell growth and motility, it is not surprising to have one or more villin/gelsolin family members affect tumorigenesis. Indeed, both gelsolin and villin are known to regulate apoptosis, cell migration, invasion, and EMT^58,59^.

Consistent with our observation with AVIL, FOXM1 has been reported to play a critical role in tumorigenesis of GBM. It can promote cell proliferation, invasion, and transform immortalized astrocytes in vitro and in vivo^14,15,17,18^. However, how is FOXM1 overexpressed in cancer is not all clear. We have accumulated the following evidence that AVIL regulates FOXM1 stability through regulating F-actin dynamics: (1) FOXM1 level was reduced with AVIL silencing, and increased with AVIL overexpression; (2) FOXM1 half-life is reduced when AVIL is silenced; (3) FOXM1 half-life is extended with AVIL overexpression; (4) FOXM1 level is enhanced with MG132, a proteasome inhibitor; (5) even with AVIL silencing, in the presence of MG132, FOXM1 remains the same as siCT; and (6) F-actin polymerization inhibitor Cytochalasin D, and depolymerization inhibitor Jasplakinolide treatment also resulted in significant reduction of FOXM1. In addition, we showed that AVIL functions upstream of FOXM1, which in turn regulates LIN28B transcription, and that AVIL mutants, which are defective in F-actin binding failed to rescue reduced LIN28B caused by AVIL silencing. Thus, our study revealed a connection between cytoskeleton and FOXM1, and established a signaling axis of AVIL/FOXM1/LIN28B/let-7.

## Methods

### Cell culture

Glioblastoma cell line U87 cells were cultured in Minimum Essential Medium Eagle (MEM), supplemented with 1 mM sodium pyruvate, 1% nonessential amino acids, 0.15% sodium bicarbonate, and 10% fetal bovine serum (FBS); T98G cells were cultured in MEM with 10% FBS; A172 cells were cultured in Dulbecco’s modified Eagle’s medium (DMEM) with 4.5 g/L glucose, and supplemented with 10% FBS; U251 cells were cultured in Roswell Park Memorial Institute medium with 5% FBS. Immortalized human astrocytes (a kind gift from Dr. Russ Pieper, University of California San Francisco) were grown in DMEM/F12 with 4.5 g/L of glucose, supplemented with 10% FBS. NSC cells were purchased from Millipore. GSC/GIC cells were gifts from Dr. Krishna Bhat, MD Anderson. They were cultured Neurobasal Media supplemented with 0.5mM L-Glutamine, B27 supplement, N2 supplement, 50 ng/ml bFGF and 50 ng/ml EGF. All cells were grown at 37 °C in 5% CO_2_–95% O_2_. All cell lines unless specified were originally obtained from ATCC. They were not further authenticated.

### Clinical samples

Fresh-frozen samples of normal brain and tumors were collected under an approved University of Virginia Institutional Review Board protocol. Tumors were macrodissected, and RNA and protein were extracted according to well-established protocols.

### AVIL overexpression and silencing

An *AVIL* cDNA clone was purchased from GeneCopoeia (GC-OG11537), and was cloned into the Retrovirus vector pQCXIH. Stable cells that overexpresss AVIL were selected via hygromycin. For siRNA treatments, siAVIL1 (targeting 5′-GCTTCTGGCAAAGGATATT-3′), siAVIL2 (targeting 5′-GCATTCCTTGCTTGTTATA-3′), and control siRNA (siGL2) were purchased from Life Technologies. RNAimax (Invitrogen) was used for siRNA transfection, which was performed according to the manufacturer’s instructions.

### PCR and real-time PCR

RNA was extracted using TRIzol reagent (Invitrogen), and quantified with Nanodrop (Thermo). cDNA was generated by AMV-RT kit (NEB), and a random hexamer primer. Real-time qPCR was carried out on the StepOne Plus system from Applied Biosystems using SYBR mix (Thermo). Primer sequences are listed in Supplementary Table 1.

### Western blotting

To measure protein levels, cell lysates were resolved by denaturing gel electrophoresis, before electrotransfer to a Protran nitrocellulose membrane. The membrane was subjected to western blot analysis with antibodies against the proteins of interest. The following antibodies and dilutions were used: rabbit anti-AVIL (1:1000; Abcam ab72210), rabbit anti-PARP (1:1000; Cell Signaling 9542), FOXM1 Antibody (G-5): (1:1000; Santa Cruz Biotechnology sc-376471), rabbit anti-Cleaved Caspase-3 (1:1000; Cell Signaling 9664), and mouse anti-GAPDH (1:10,000; Ambion Am4300).

### FISH

DNA probes for fluorescence in situ hybridization (FISH) were labeled by nick translation with SpectrumGreen or SpectrumRed 2′-deoxyuridine-5′-triphospahte (Abbott).

Cells were grown in 8-chamber slides and fixed with methanol:acetone = 1:1 fixation solution for 10 min at 4 °C. BAC Fish clone, RP11-143123 was purchased from BACPAC.

### Immunohistochemistry

Immunohistochemistry for AVIL was performed on a robotic platform (Ventana discover Ultra Staining Module, Ventana Co., Tucson, AZ, USA). Tissue sections were deparaffinized using EZ Prep solution (Ventana). A heat-induced antigen retrieval protocol set for 64 min was carried out using a TRIS–ethylenediamine tetracetic acid (EDTA)–boric acid pH 8.4 buffer (Cell Conditioner 1). Endogenous peroxidases were blocked with peroxidase inhibitor (CM1) for 8 min before incubating the cells with AVIL antibody (Abcam, Ab72210) at 1:100 dilution for 60 min at room temperature. Antigen-antibody complex was then detected using DISCOVERY anti-rabbit HQ HRP detection system and DISCOVERY ChromoMap DAB Kit (Ventana Co.). All the slides were counterstained with hematoxylin subsequently; they were dehydrated, cleared, and mounted for the assessment.

For cleaved caspase3, and Ki67, A heat-induced antigen retrieval protocol set for 64 min was carried out using a TRIS–EDTA–boric acid pH 8.4 buffer (CC1). Endogenous peroxidases were blocked with peroxidase inhibitor before incubating the section with cleaved Caspase 3 antibody (Cell Signaling, Cat #9661) at 1:400 dilution for 1 h at room temperature, antigen-antibody complex was then detected using DISCOVERY anti-rabbit HQ HRP detection system and DISCOVERY ChromoMap DAB Kit (Ventana Co.) The slide was next incubated with Ki67 antibody (Abcam, Ab16667) at 1:300 for 1 h at room temperature and then incubated with DISCOVERY anti-rabbit HQ HRP detection system and the DISCOVERY Purple kit. All slides were counterstained with hematoxylin subsequently; they were dehydrated, cleared, and mounted for the assessment.

### Semiquantitative analysis of AVIL Immunohistochemistry

Archival Formalin-Fixed, Paraffin-Embedded (FFPE) specimens were processed for immunohistochemistry with the anti-AVIL antibody. Immunohistochemical scoring was performed by a neuropathologist blinded to the diagnosis, histologic grade, and IDH1 mutation status of the tumor. Scoring was performed on a scale of 0–3, where 0 = 0% cells positive, 1 => 0% <10% cells positive, 2 => 10% <50% cells positive, and 3 => 50% cells positive.

### Immunofluorescence and confocal microscopy

For GFP-AVIL, U87 cells were transfected with GFP-AVIL expressing plasmid, and seeded on chamber slide and subsequently co-fixed with 4% PFA and 0.5% Triton-X 100 for 10 min at 37 °C and blocked with 5% BSA in PBS. Cells were then incubated with Texas Red™-X Phalloidin (ThermoFisher Scientific) for 45 min at room temperature, washed with PBS, and stained with DAPI. Cells were subsequently examined under a Zeiss LSM 510 laser scanning fluorescence confocal microscope at 400x nominal magnification. For other immunofluorescence experiments, fixed cells were typically blocked and incubated overnight with primary antibodies. Subsequently, the slides were washed with PBS, incubated with fluorescence-conjugated secondary antibodies for 1 h at room temperature, before mounted and examined by Zeiss LSM 510 laser scanning fluorescence confocal microscope.

### Cell migration and invasion assay

The effect of AVIL on cell migration was assayed by a wound-healing assay. Briefly, cells were cultured to confluency. A wound was created by scraping the cells using a 10 ul plastic pipette tip, and the medium was replaced with fresh medium. Images were captured immediately after the scratch, and again 6 h later. Cell migration was qualitatively assessed by the size of the gap within the confluent monolayer culture at the end of the experiment. Eight gaps were measured. For siRNA experiment, the measurement took place around 48–72 h after transfection.

The effect of AVIL on cell invasion was assessed by a transwell invasion assay. siAVIL or siGL2 transfected glioblastoma cells (1 × 10^5^), after 72 h transfection were suspended in 300 μL 0.1% FBS medium, and added to the upper chamber of the wells. The lower chamber contained 600 μL of 10% FBS medium. The plate was kept in air with 5% CO_2_ for 8 h at 37 °C. The cells on the upper membrane surface were then mechanically removed. The cells that had migrated to the lower side of the collagen IV–coated membrane were fixed and stained with 0.1% crystal violet. Migrated cells were counted in five randomly chosen fields under a microscope, and the average number of these cells per field was calculated.

### Live-cell imaging

The cellular movement was analyzed by live-cell imaging. Briefly, A172 were plated to 40–60% confluency in DMEM + 10% FBS without Phenol Red, followed by transfection with siGL2 or siAVIL. 2 h prior to start of imaging media was supplemented with 1 µM SiR-DNA (Cytoskeleton) dye. 24 h after siRNA transfection images were collected on a Zeiss Axio-observer-Z1 epifluorescent microscope in humidified chamber in 5% CO_2_ environment, at 37 °C every 20 min over the period of 24 h. Resulting movies were processed ImageJ (https://imagej.nih.gov/ij/), and cell movement was tracked semi-automatically based on DNA staining by TrackMate plugin for ImageJ.

Analysis of the movement of GSC11 glioma stem cells was done similarly as described above with following modifications. 2 h after the transfection with either siGL2 or siAVIL GSC11 cells were transferred to the 24-well clear bottom plate in media containing 50% MatriGel matrix (Corning) supplemented with 1 µM SiR-DNA. Images were collected similarly to described above, starting 6 h after transfection. Subsequent analysis was performed utilizing TrackMate plugin in ImageJ.

For the experiment depicted in Fig. S14, A172 cells expressing mCherry-Lifeact were plated onto Nunc™ Lab-Tek™ II Chambered Coverglass with 1.5 Borosilicate glass (ThermoFisher Scientific) previously coated with 1 µg/ml fibronectin and subsequently transfected with siGL2 or siAVIL at ~40% confluency. 24 h after transfection media was replaced with DMEM + 10% FBS without Phenol Red (Gibco), supplemented with 20 mM HEPES buffer. Movies were collected for 10 min by inverted confocal microscope (Zeiss, Jena, Germany) using a Nakagawa spinning disc and with frames taken every 2 s. Following movies were processed and analyzed in ImageJ.

### Cell morphology assessment

All materials were from Life Technologies unless otherwise indicated. In-house-made dishes with glass coverslip bottoms were pre-adsorbed with 2 µg/ml fibronectin overnight. Cells were stained with 5 µM DiI in OptiMEM for 15 min, then rinsed with PBS and returned to normal growth medium until the experiment. Cells were seeded on glass-bottomed dishes in serum-free, CO2-independent medium (CCM1, Hyclone), and cultured for 1.5 h. Each sample was imaged live on an Olympus Fluoview 1000 laser scanning confocal microscope with a 10× (0.3 NA) objective. The samples were maintained at 37 °C with a stage heater. The DiI-stained cells were excited using the 543 nm line of a HeNe laser. Images were acquired at a resolution of 0.795 µm/pixel. Settings were adjusted to minimize photodamage.

Cell area measurements were automated using custom MATLAB scripts. Briefly, cell images were subjected to an interactive threshold, resulting in silhouettes, which were then automatically quantified using MATLAB’s built-in region properties function. Cell areas (µm^2^) of the control and AVIL knock-down groups were compared using the Rank-Sum test for non-normally distributed data. The sample sizes for the control and knock-down groups were 55 and 51 cells, respectively.

### Tumor formation in vivo

The mouse work was performed under the study protocol 4234 as approved by the University of Virginia Institutional Animal Care and Use Committee. Immunocompromised SCID/NCr BALB/c adult male mice (6–8 weeks old) were used. All animals were housed in sterilized plastic cages under specific pathogen-free conditions, at 22 °C, 12/12 light/dark cycle, 55% humidity. U251 or GSC11 cells were transfected with control shRNA, or shAVIL1 for 12 h. The transfected cells were then counted, and 3 × 10^5^ were stereotactically (Stoelting) implanted into the right corpus striatum of the mice. Cerebral magnetic resonance imaging was performed on anesthetized mice at 4 weeks post implantation. Ten to fifteen minutes before scanning, 30 ul of Magnevist brand gadopentetate dimeglumine was injected intraperitoneally. T1-weighted serial coronal images of each brain were acquired at 1 mm intervals with a 5 × 5 mm field, and a 256 × 256 pixel resolution. For image analysis and tumor volume quantification, a luminosity histogram was first generated for a selected area of the left cerebrum that was grossly tumor-free. This served as an internal control. Pixel luminosity mean and SD were noted. Histogram generation was repeated on a similar selection from the right cerebrum that contained all enhanced tumor. Pixels in the right cerebrum, greater than two SDs above the left cerebrum control luminosity mean, were recorded as representing enhanced tumor for a given image. This procedure was then repeated for all the images showing an enhanced tumor for a given brain, thus generating a sum of enhanced tumor pixels for each brain. Tumor volume relates to tumor pixels in a linear manner, and was calculated based on the image acquisition, interval distance, and resolution.

Astrocytes stably expressing AVIL or an empty vector were injected subcutaneously into the flanks of NIH-III Nude mice. Around 2 million cells were used per injection. The animals were monitored twice a week. For drug treatment, 1.5 million U87 cells were injected subcutaneously into the flanks of NIH-III Nude mice.

### Protein purification

The sequence of human Advillin (AVIL) was cloned to pMAL-c4x vector, modified to express 6xHis tag on MBP N-termini, and the corresponding plasmid was transformed into BL21 (DE3) cells. Expression of 6xHis-MBP-AVIL was induced at OD_600_ ≈ 0.7 by addition of 1 mM IPTG and continued for 18 h at 23 °C. Harvested cells were subsequently resuspended in 50 mM Tris-HCl pH 7.4, 300 mM NaCl, 10 mM Imidazole, 5 mM β-mercaptoethanol (Resuspension Buffer) supplemented with Complete Protease Inhibitor Cocktail (Roche), lysed in Emulsiflex C3 (AVESTIN) and cell debris was removed by centrifugation. 6xHis-MBP-AVIL was immobilized on Nickel NTA agarose (Gold Biotechnology) followed by an extensive wash (>50 column volumes) with 50 mM Tris-HCl pH 7.4, 100 mM NaCl, 40 mM Imidazole, 5 mM β-mercaptoethanol. Protein was eluted with 50 mM Tris-HC; pH 7.4, 100 mM NaCl, 250 mM Imidazole, 5 mM β-mercaptoethanol. After overnight incubation with TEV protease 6xHis-MBP-tag was removed by NiNTA and AVIL was further purified by size exclusion on HiLoad 16/600 Superdex 200 pg column, previously equilibrated to 20 mM HEPES, pH 7.4, 100 mM NaCl, 2 mM tris(2-carboxyethyl)phosphine (TCEP). Recombinant AVIL was flash-frozen in liquid nitrogen and stored at −80 °C.

### Actin preparation, and binding assay

Actin was purified from rabbit muscle acetone powder and stored in ATP-G-buffer on ice. For actin-binding experiment 20 µM G-actin was supplemented with 100 mM KCl and 2 mM MgCl_2_ to induce actin polymerization and incubated for 1 h at room temperature. F-actin was subsequently diluted to final concentration of 2 µM and incubated with 2 µM AVIL wild-type or mutants for 1 h at room temperature in binding/bundling buffer (20 mM HEPES pH 7.4, 100 mM KCl, 2 mM EGTA) followed by 30-min centrifugation at 100,000 RCF. The supernatant was then collected, and the remaining pellet was resuspended in 1x Sample Buffer. Samples were analyzed by SDS-PAGE followed by silver staining.

### Statistics and reproducibility

All the experiments were repeated at least three times unless otherwise noted. All quantitative data were presented as the mean ± SEM (standard error of the mean) or the mean ± SD (standard deviation) as indicated of at least three independent experiments by Student’s *t-*test for between-group differences. The *P* < 0.05 was considered statistically significant.

### Reporting summary

Further information on research design is available in the Nature Research Reporting Summary linked to this article.

## Supplementary information

Supplementary Information

Description of Additional Supplementary Files

Supplementary Movie 1

Supplementary Movie 2

Reporting Summary

## Data Availability

Full scans of the gels and blots are available in Supplementary Fig. 21. The Raw and processed RNA-Sequencing data from this study have been submitted to the NCBI Gene Expression Omnibus (GEO; http://www.ncbi.nlm.nih.gov/geo/) under accession number GSE64032 [https://www.ncbi.nlm.nih.gov/geo/query/acc.cgi?acc=GSE64032]. Microarray data for this study has also been deposited onto the GEO database, GSE95164 [https://www.ncbi.nlm.nih.gov/geo/query/acc.cgi?acc=GSE95164]. All relevant data are available from the corresponding author upon reasonable request. All the other data supporting the findings of this study are available within the article and its supplementary information files and from the corresponding author upon reasonable request. A reporting summary for this article is available as a Supplementary Information file.

## Code availability

The custom codes in MATLAB are available by authors upon request.

## References

[1] Castro MG (2003). Current and future strategies for the treatment of malignant brain tumors. Pharm. Ther..

[2] King GD (2005). Gene therapy and targeted toxins for glioma. Curr. Gene Ther..

[3] Stupp R (2005). Radiotherapy plus concomitant and adjuvant temozolomide for glioblastoma. N. Engl. J. Med.

[4] Weinstein IB (2002). Cancer. Addiction to oncogenes–the Achilles heal of cancer. Science.

[5] Vivanco I (2014). Targeting molecular addictions in cancer. Br. J. Cancer.

[6] Luo J, Solimini NL, Elledge SJ (2009). Principles of cancer therapy: oncogene and non-oncogene addiction. Cell.

[7] Druker BJ (2001). Efficacy and safety of a specific inhibitor of the BCR-ABL tyrosine kinase in chronic myeloid leukemia. N. Engl. J. Med.

[8] Paik S, Kim C, Wolmark N (2008). HER2 status and benefit from adjuvant trastuzumab in breast cancer. N. Engl. J. Med.

[9] Bollag G (2010). Clinical efficacy of a RAF inhibitor needs broad target blockade in BRAF-mutant melanoma. Nature.

[10] Network TC (2013). Corrigendum: comprehensive genomic characterization defines human glioblastoma genes and core pathways. Nature.

[11] Yilmaz M, Christofori G (2009). EMT, the cytoskeleton, and cancer cell invasion. Cancer Metastasis Rev..

[12] Wang L (2015). Expression of cortactin in human gliomas and its effect on migration and invasion of glioma cells. Oncol. Rep..

[13] Zhang S, Qi Q (2015). MTSS1 suppresses cell migration and invasion by targeting CTTN in glioblastoma. J. Neurooncol.

[14] Liu M (2006). FoxM1B is overexpressed in human glioblastomas and critically regulates the tumorigenicity of glioma cells. Cancer Res.

[15] Zhang N (2011). FoxM1 promotes beta-catenin nuclear localization and controls Wnt target-gene expression and glioma tumorigenesis. Cancer Cell.

[16] Zhang Y (2008). FoxM1B transcriptionally regulates vascular endothelial growth factor expression and promotes the angiogenesis and growth of glioma cells. Cancer Res.

[17] Dai B (2007). Aberrant FoxM1B expression increases matrix metalloproteinase-2 transcription and enhances the invasion of glioma cells. Oncogene.

[18] Dai B (2010). FoxM1B regulates NEDD4-1 expression, leading to cellular transformation and full malignant phenotype in immortalized human astrocytes. Cancer Res.

[19] Wang XR (2013). Overexpressed let-7a inhibits glioma cell malignancy by directly targeting K-ras, independently of PTEN. Neuro Oncol..

[20] Xie Z (2016). Fusion transcriptome profiling provides insights into alveolar rhabdomyosarcoma. Proc. Natl Acad. Sci. USA.

[21] Marks PW, Arai M, Bandura JL, Kwiatkowski DJ (1998). Advillin (p92): a new member of the gelsolin/villin family of actin regulatory proteins. J. Cell Sci..

[22] Cerami E (2012). The cBio cancer genomics portal: an open platform for exploring multidimensional cancer genomics data. Cancer Disco..

[23] Gao J (2013). Integrative analysis of complex cancer genomics and clinical profiles using the cBioPortal. Sci. Signal.

[24] Reifenberger G (1996). Refined mapping of 12q13-q15 amplicons in human malignant gliomas suggests CDK4/SAS and MDM2 as independent amplification targets. Cancer Res.

[25] Madhavan S (2009). Rembrandt: helping personalized medicine become a reality through integrative translational research. Mol. Cancer Res.

[26] Ceccarelli M (2016). Molecular profiling reveals biologically discrete subsets and pathways of progression in diffuse glioma. Cell.

[27] Cancer Genome Atlas Research, N. (2015). Comprehensive, integrative genomic analysis of diffuse lower-grade gliomas. N. Engl. J. Med..

[28] Candolfi M (2007). Intracranial glioblastoma models in preclinical neuro-oncology: neuropathological characterization and tumor progression. J. Neurooncol..

[29] Bradley CA (2017). Glioblastoma: stem cells - masters of their fates. Nat. Rev. Cancer.

[30] Bao S (2006). Glioma stem cells promote radioresistance by preferential activation of the DNA damage response. Nature.

[31] Li X (2014). Short laminin peptide for improved neural stem cell growth. Stem Cells Transl. Med.

[32] Parsons DW (2008). An integrated genomic analysis of human glioblastoma multiforme. Science.

[33] Cancer Genome Atlas Research, N. (2008). Comprehensive genomic characterization defines human glioblastoma genes and core pathways. Nature.

[34] Brennan CW (2013). The somatic genomic landscape of glioblastoma. Cell.

[35] Walsh AM (2015). Sprouty2 drives drug resistance and proliferation in glioblastoma. Mol. Cancer Res.

[36] Kim J (2010). Functional genomic screen for modulators of ciliogenesis and cilium length. Nature.

[37] Ridley AJ (2011). Life at the leading edge. Cell.

[38] Zhai L, Zhao P, Panebra A, Guerrerio AL, Khurana S (2001). Tyrosine phosphorylation of villin regulates the organization of the actin cytoskeleton. J. Biol. Chem..

[39] Chen X (2013). The forkhead transcription factor FOXM1 controls cell cycle-dependent gene expression through an atypical chromatin binding mechanism. Mol. Cell Biol..

[40] Wang Z (2018). BART: a transcription factor prediction tool with query gene sets or epigenomic profiles. Bioinformatics.

[41] Shi M, Cui J, Xie K (2013). Signaling of miRNAs-FOXM1 in cancer and potential targeted therapy. Curr. Drug Targets.

[42] Viswanathan SR (2009). Lin28 promotes transformation and is associated with advanced human malignancies. Nat. Genet..

[43] Liang L (2010). MicroRNA-125b suppressesed human liver cancer cell proliferation and metastasis by directly targeting oncogene LIN28B2. Hepatology.

[44] Nguyen LH (2014). Lin28b is sufficient to drive liver cancer and necessary for its maintenance in murine models. Cancer Cell.

[45] Kugel S (2016). SIRT6 suppresses pancreatic cancer through control of Lin28b. Cell.

[46] Busch B (2016). The oncogenic triangle of HMGA2, LIN28B and IGF2BP1 antagonizes tumor-suppressive actions of the let-7 family. Nucleic Acids Res..

[47] Dunn GP (2012). Emerging insights into the molecular and cellular basis of glioblastoma. Genes Dev..

[48] American Brain Tumor Association. (2014).

[49] Marino-Enriquez A, Dal Cin P (2013). ALK as a paradigm of oncogenic promiscuity: different mechanisms of activation and different fusion partners drive tumors of different lineages. Cancer Genet.

[50] Morris SW (1994). Fusion of a kinase gene, ALK, to a nucleolar protein gene, NPM, in non-Hodgkin’s lymphoma. Science.

[51] Soda M (2007). Identification of the transforming EML4-ALK fusion gene in non-small-cell lung cancer. Nature.

[52] Chen Y (2008). Oncogenic mutations of ALK kinase in neuroblastoma. Nature.

[53] van Gaal JC (2012). Anaplastic lymphoma kinase aberrations in rhabdomyosarcoma: clinical and prognostic implications. J. Clin. Oncol..

[54] Shaw AT (2011). Effect of crizotinib on overall survival in patients with advanced non-small-cell lung cancer harbouring ALK gene rearrangement: a retrospective analysis. Lancet Oncol..

[55] Rao J (2017). Advillin acts upstream of phospholipase C 1 in steroid-resistant nephrotic syndrome. J. Clin. Invest.

[56] Asch HL (1996). Widespread loss of gelsolin in breast cancers of humans, mice, and rats. Cancer Res.

[57] Wang Y (2008). A novel role for villin in intestinal epithelial cell survival and homeostasis. J. Biol. Chem..

[58] Khurana S (2008). Autotaxin and lysophosphatidic acid stimulate intestinal cell motility by redistribution of the actin modifying protein villin to the developing lamellipodia. Exp. Cell Res..

[59] Nag S, Larsson M, Robinson RC, Burtnick LD (2013). Gelsolin: the tail of a molecular gymnast. Cytoskeleton (Hoboken).

