# Supplementary material for: A cytoskeleton regulator AVIL drives tumorigenesis in glioblastoma
Source: Nat Commun. 2020 Jul 10;11:3457. doi: 10.1038/s41467-020-17279-1 (PMC7351761; doi:10.1038/s41467-020-17279-1)
Supplement: Supplementary file 1 — Supplementary Information [file 41467_2020_17279_MOESM1_ESM.pdf]

SUPPLEMENTARY INFORMATION

A Cytoskeleton Regulator AVIL Drives Tumorigenesis in Glioblastoma

NCOMMS-19-15773B

Xie et al.,

Supplemental Figure 1.

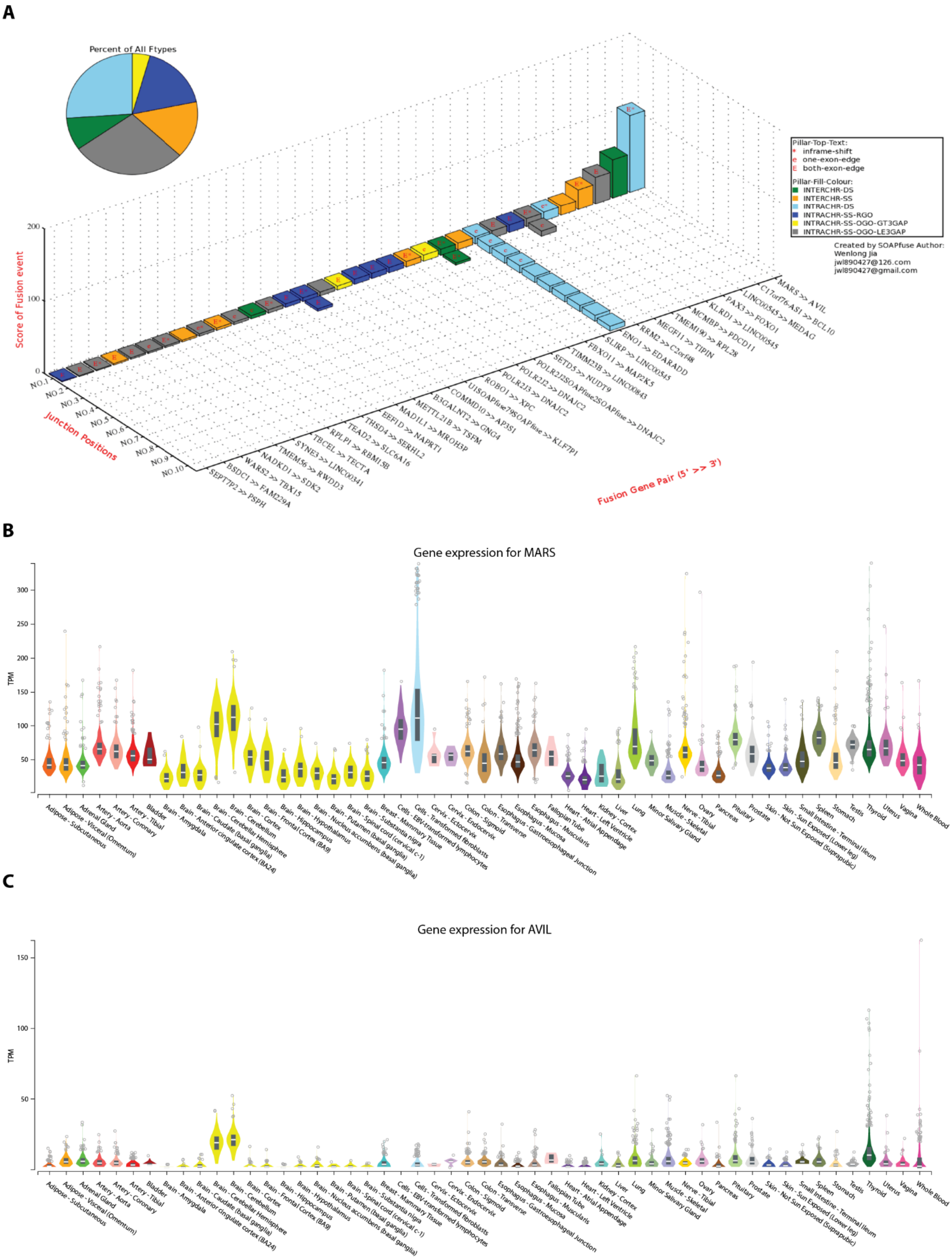

Supplementary Figure 1. MARS-AVIL fusion and their parental gene expression. (A) Landscape of fusion RNAs in RH30 RNA-sequencing. *MARS-AVIL* fusion has the highest number of reads. (B) *MARS* is expressed ubiquitously. (C) *AVIL* is hardly expressed in most tissues. The analysis is extracted from GTEx portal.

Supplemental Figure 2.

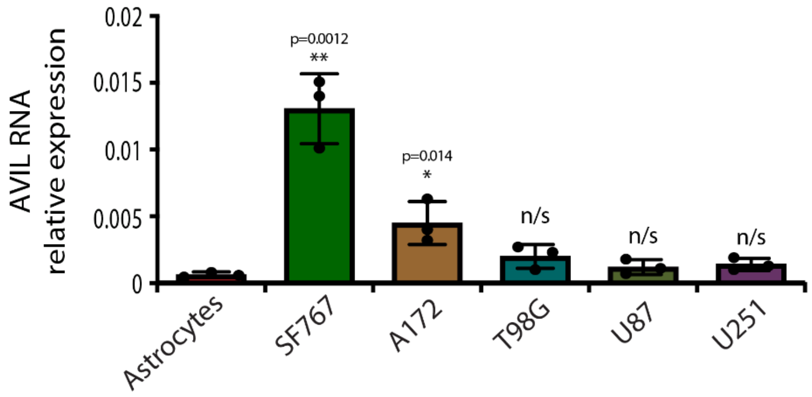

Supplementary Figure 2. AVIL RNA expression. qRT-PCR measuring *AVIL* mRNA level in GBMs, and control astrocytes. *AVIL* RNA expression was normalized against that of *GAPDH* (two-sided Student's t test). Data are presented as mean values +/- SD. * $p<0.05$, ** $p<0.01$

Supplemental Figure 3.

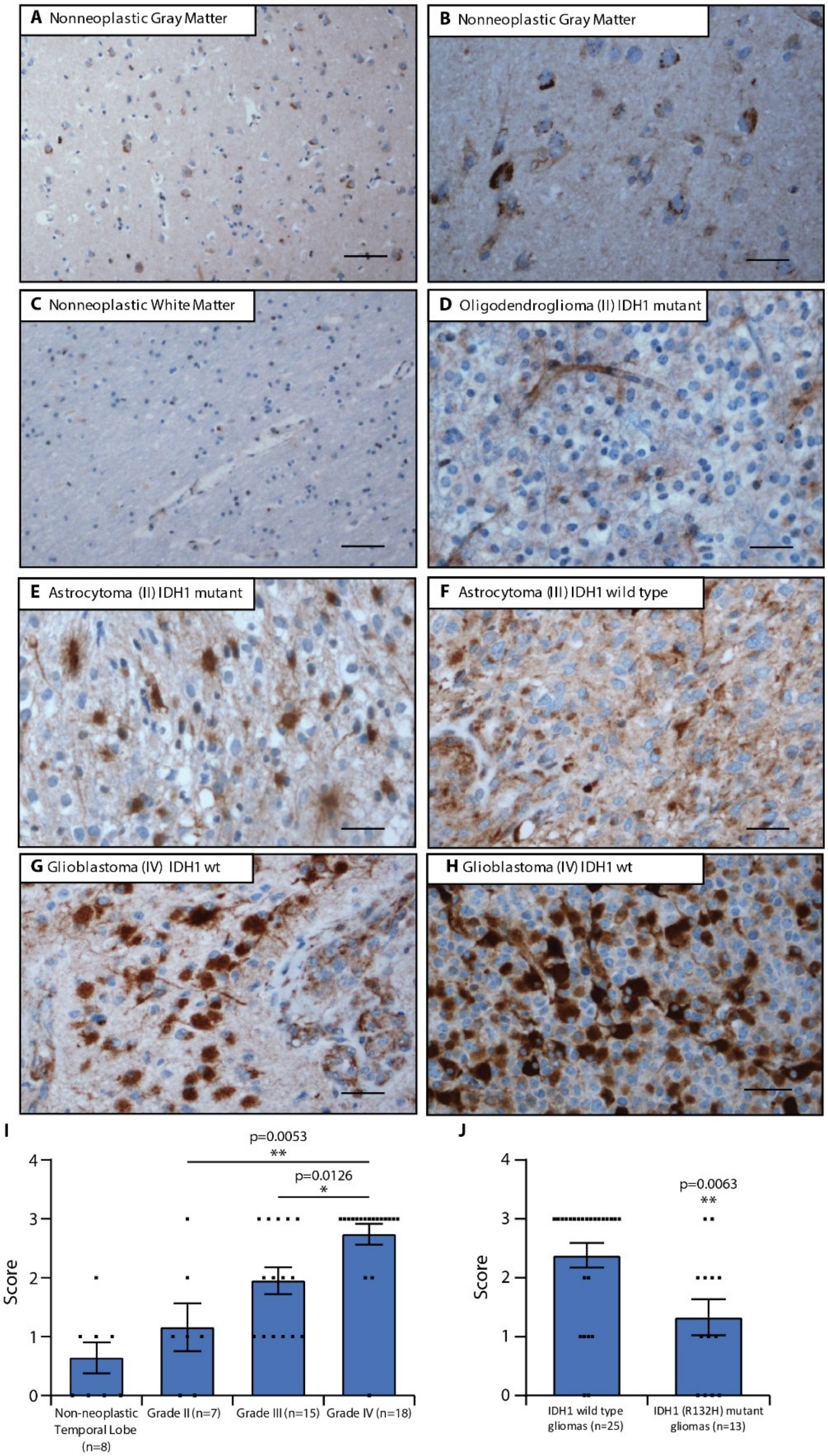

Supplementary Figure 3. Immunohistochemical staining for AVIL protein expression. (A, B) Expression in normal human cerebral cortical gray matter is largely restricted to a subpopulation of pyramidal neurons, with a distinct perinuclear punctate localization. There is also a low expression in neuropil. (C) Normal white matter demonstrates little immunoreactivity. (D) Oligodendrogliomas generally show little or no tumor cell labeling. (E, F) Astrocytomas show moderate immunolabeling in a subset of neoplastic cells. (G, H). Glioblastomas typically show intense cytoplasmic advillin immunolabeling in a majority of tumor cells Scale bars represent 50 µm in A, C; and 25 µm in B, D, E, F, G. (I) Semiquantitative analysis of AVIL immunohistochemistry in human gliomas reveals significant correlation with Histologic Grade. Using unpaired T-tests, significant differences are detected between Grade IV and each other group (comparison of grade IV and grade III p=0.0126; comparison of grade IV and grade II p=0.0072) (J) Semiquantitative analysis of AVIL immunohistochemistry with regard to IDH status. Significant with a two-tailed P-value of 0.0063. Data are presented as mean values +/- SEM in I and J.

Supplemental Figure 4.

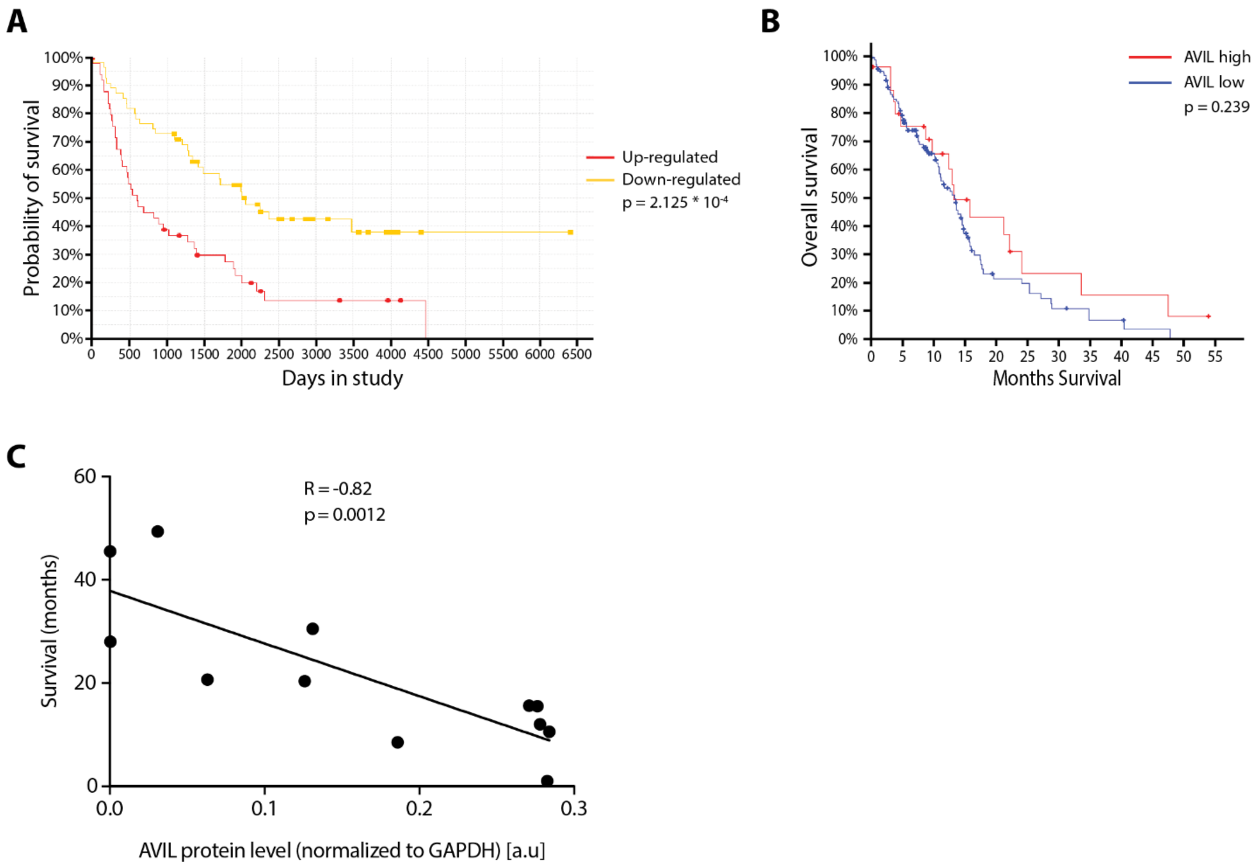

Supplementary Figure 4. Clinical correlation between AVIL and glioma patient outcome. (A) A two-class model stratified by *AVIL* expression in 104 glioma cases that had microarray probe 1568706 signal. Higher *AVIL* expression correlates with poor patient prognosis ($p=2x10^{-4}$) (two-sided log-rank test). (B) Absence of significant correlation between *AVIL* RNA expression and GBM patient survival was observed (p=0.239) (two-sided log-rank test). (C) Inverse correlation between AVIL protein level expression and GBM patient survival. (R=-0.82, p=0.0012) (Pearson correlation).

Supplemental Figure 5.

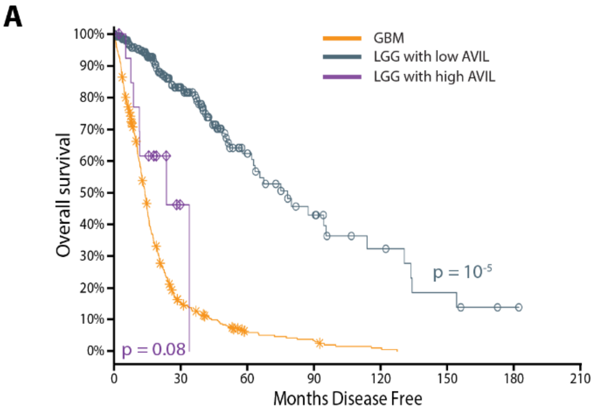

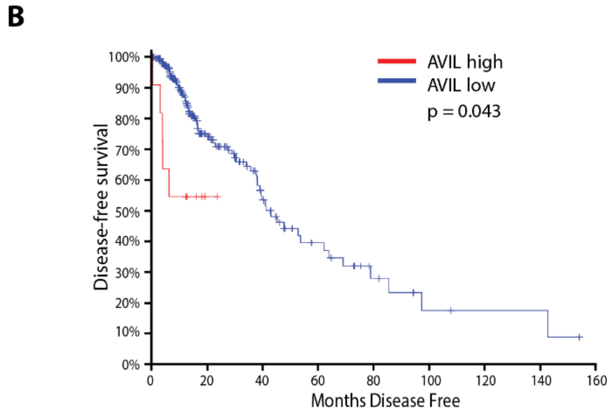

Supplementary Figure 5. Clinical correlation between AVIL and lower grade gliomas (LGG) patient outcome. (A) A two-class model stratified by *AVIL* expression in 283 LGGs. The overall survival for the group with higher *AVIL* expression is significantly shorter than the group with lower *AVIL* expression (p=1E-5), and not significant different from the survival of GBM patients (p=0.08) (two-sided log-rank test). (B) Disease-free survival between the two groups was also significantly different ($p<0.05$) (two-sided log-rank test).

Supplemental Figure 6.

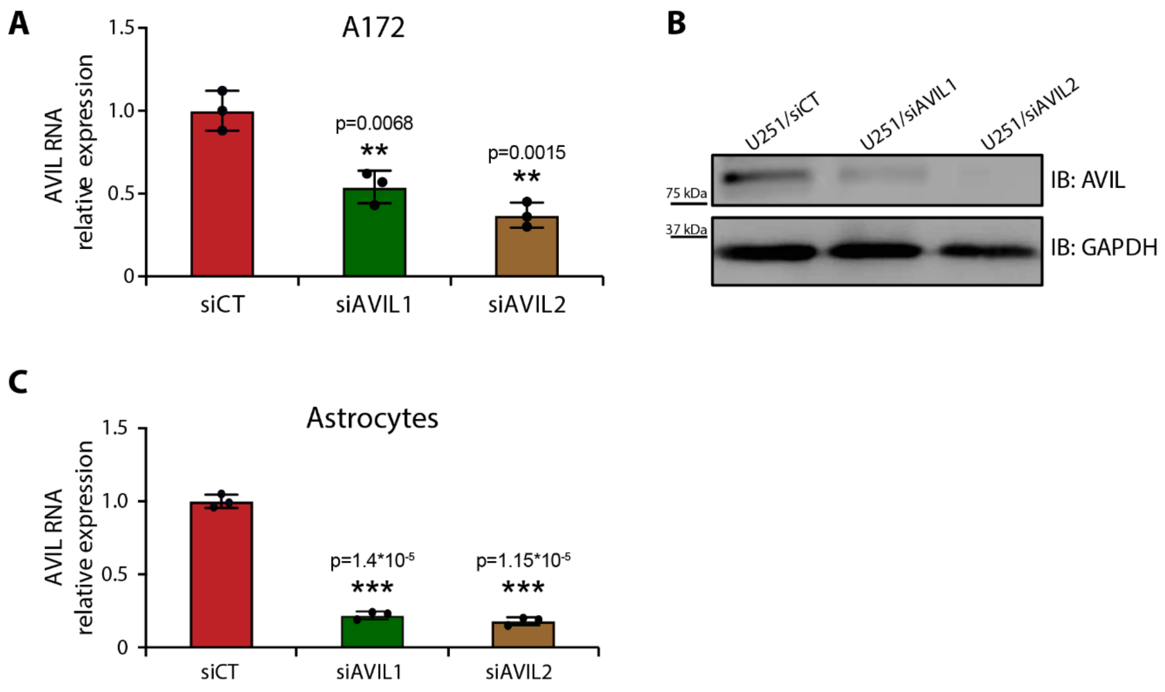

Supplementary Figure 6. siRNAs targeting AVIL resulted in its reduced expression. (A) A172 cells was transfected with siAVIL1, siAVIl2, or siCT. *AVIL* level was measured by qRT-PCR, normalized to *GAPDH*, and further normalized to that in siCT. P value was calculated by standard two-tailed t-test. (B) Western blot analysis measuring AVIL protein in U251 cells transfected with siAVIL1, siAVIl2, or siCT. (C) qRT-PCR measuring *AVIL* RNA in astrocytes cells transfected with siAVIL1, siAVIL2, or siCT. P value was calculated by standard two-tailed t-test. * p<0.05, ** p<0.01, *** p<0.001. Data are presented as mean values +/- SD in A and C.

Supplemental Figure 7.

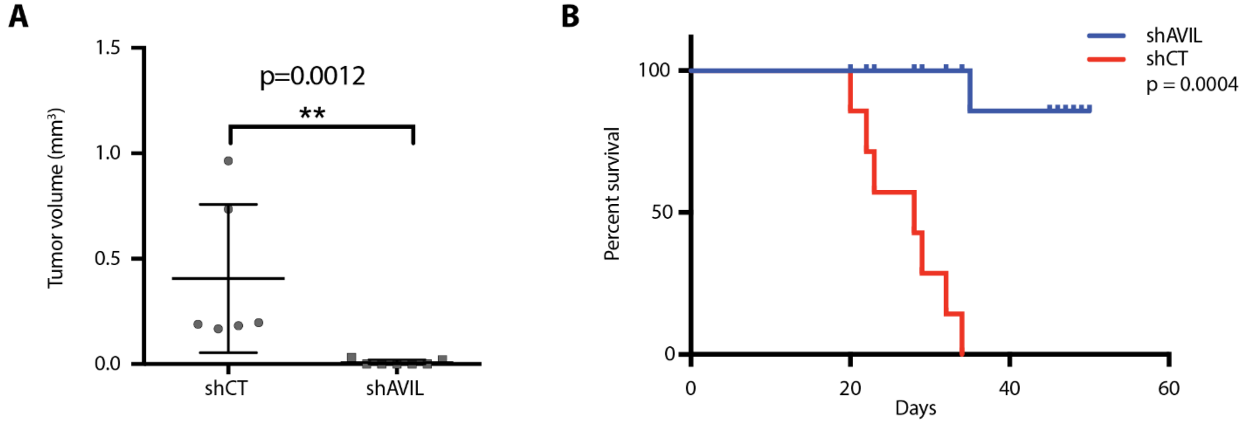

Supplementary Figure 7. AVIL is crucial for glioblastoma initiation *in vivo*. (A) Mice were intracranially injected with U251 cells stably expressing shCT or shAVIL. shAVIL group has almost no tumors (two-sided Student's t test). (B) Percent of survival of the animals was plotted according to Kaplan-Meier analysis. shAVIL group had much longer survival. p=0.0004 (two-sided log-rank test). Data are presented as mean values +/- SD in A. shCT group n=6, shAVIL group n=7.

Supplemental Figure 8.

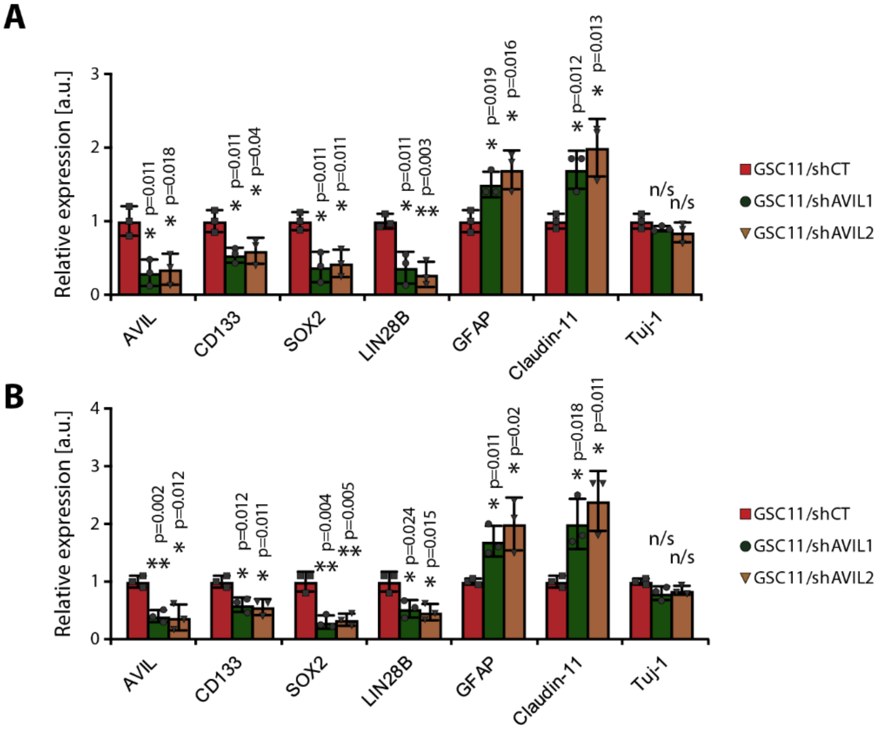

Supplementary Figure 8. Silencing AVIL resulted in recued stemness, and enhanced differentiation markers. qRT-PCR measuring *AVIL,* stemness, and various differentiation markers in GSC11 (A) or GSC627 (B) transfected with shAVIL1, shAVIL2, or control shCT. Various transcripts were normalized against that of *GAPDH*, and further normalized to the level in shCT group. P value was calculated by standard two-tailed t-test. * p<0.05, ** p<0.01, *** p<0.001. Data are presented as mean values +/- SD.

Supplemental Figure 9.

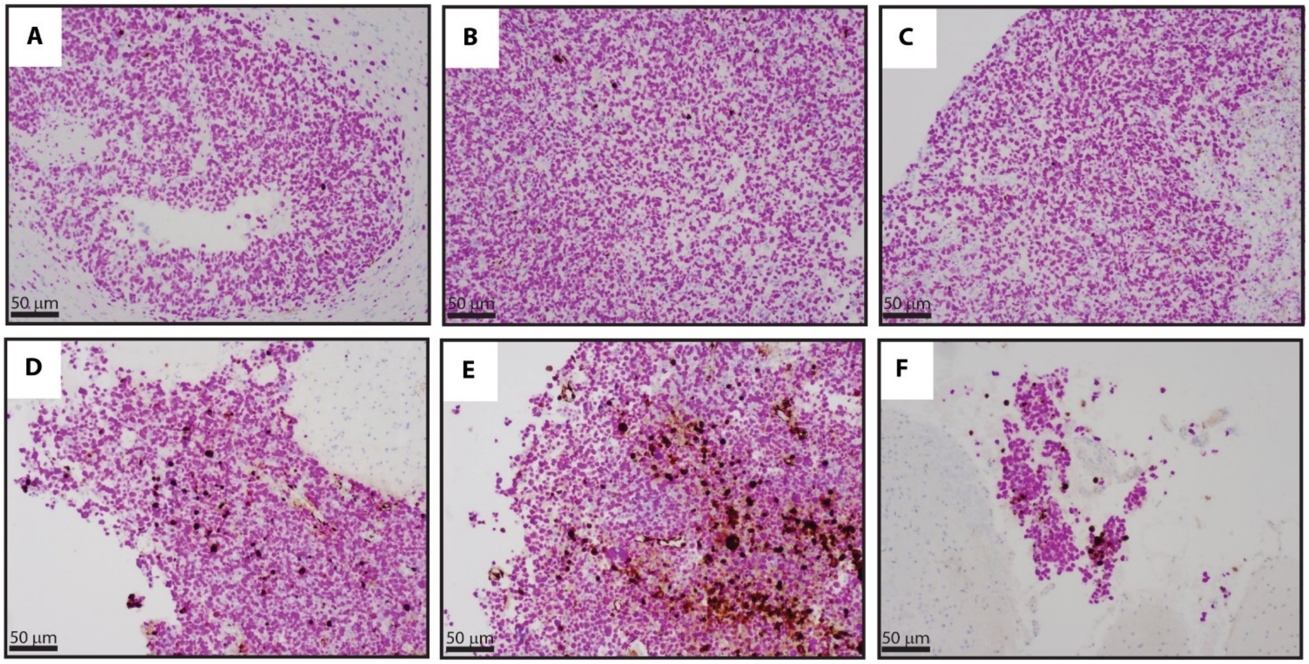

Supplementary Figure 9. AVIL silenced GSC11 intracranial xenografts have more apoptosis. Ki67 (pink) and cleaved Caspase3 (brown) staining on GSC11 intracranial xenografts. (A-C) shCT group. (D-F) shAVIL group.

Supplemental Figure 10.

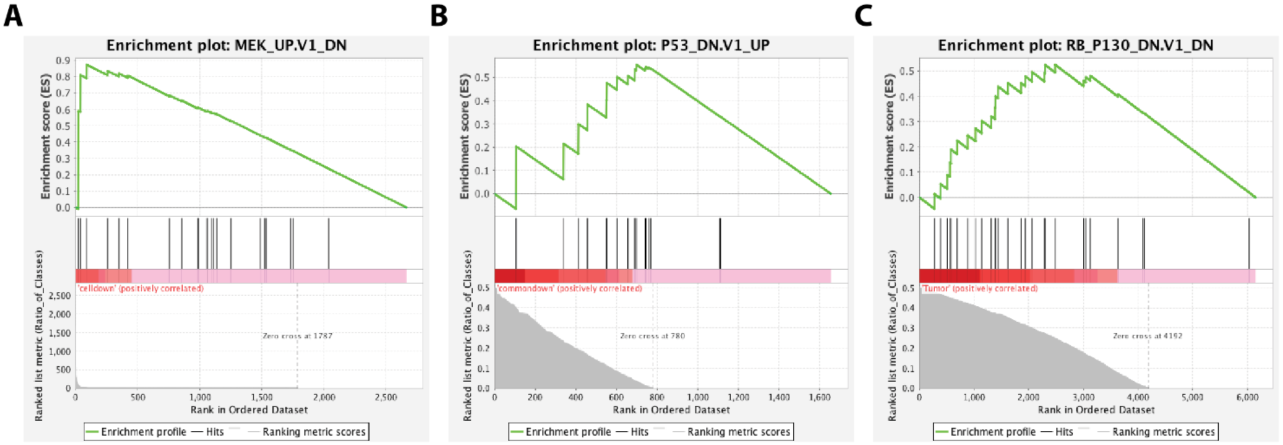

Supplementary Figure 10. Gene Set Enrichment Analysis (GESA) revealed significant enrichment of MAPK (A), TP53 (B), and RB (C) pathways with AVIL overexpression in astrocytes.

Supplemental Figure 11.

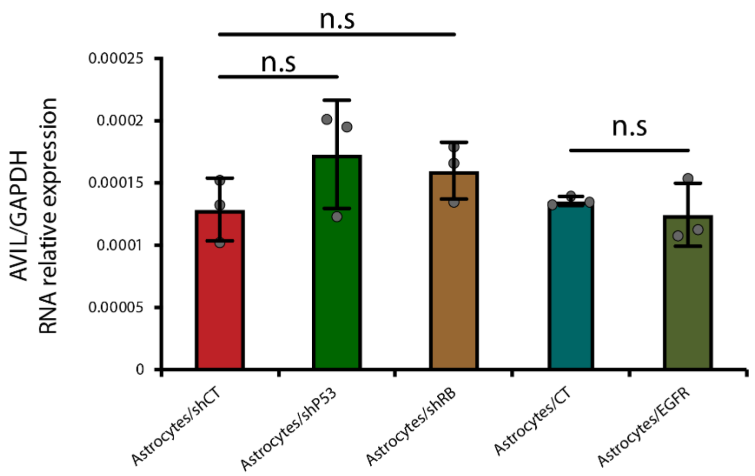

Supplementary Figure 11. Astrocytes transfected with shP53, ShRB, or EGFRvIII mutant had no effect on *AVIL* expression. *AVIL* level was measured by qRT-PCR, and normalized to *GAPDH*. Data are presented as mean values +/- SD.

Supplemental Figure 12.

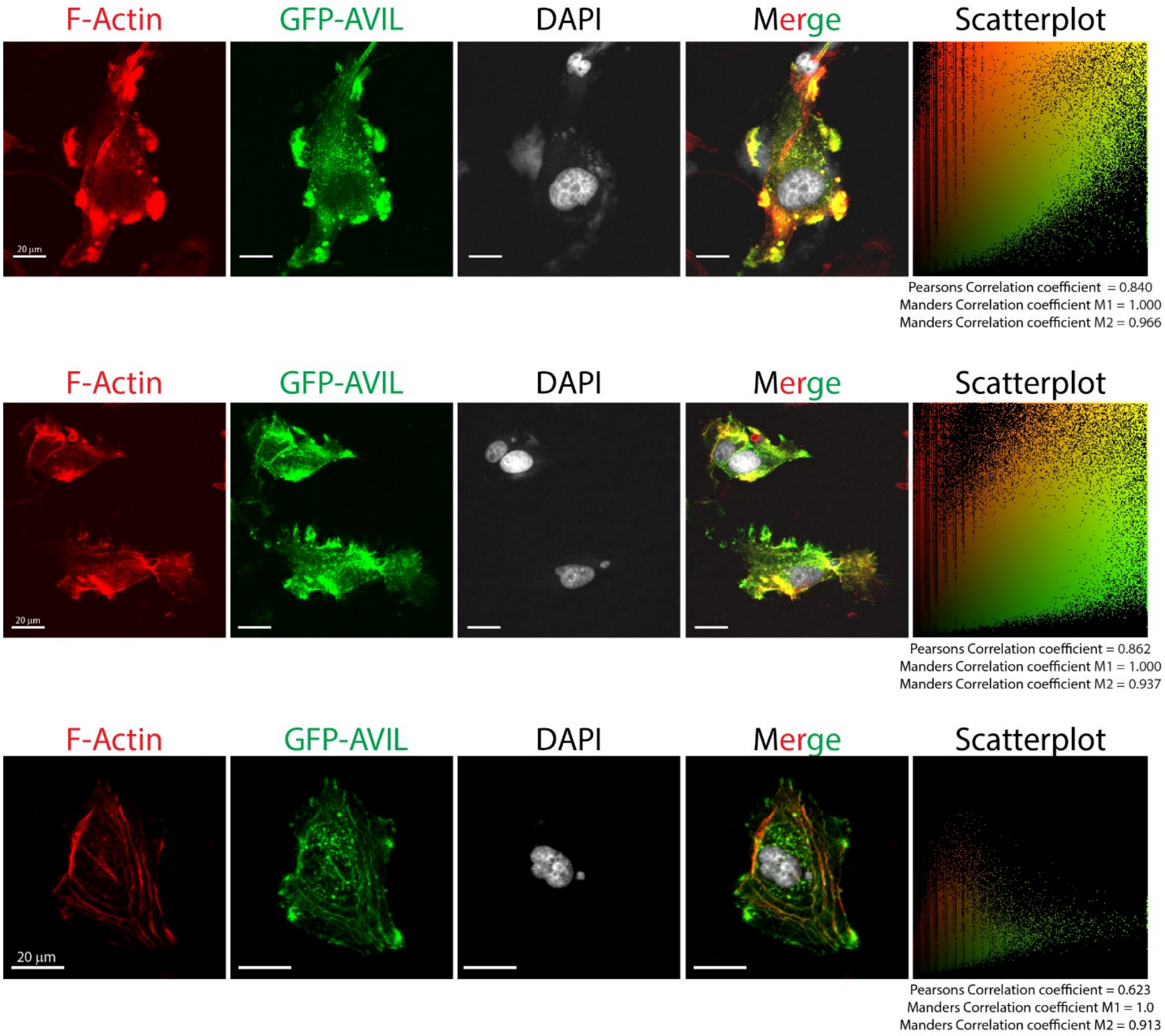

Supplementary Figure 12. AVIL co-localizes with F-actin. U87 cells stably expressing GFP-AVIL (green) were stained for F-actin (Phalloidin, red) and DNA (DAPI, grey), showing co-localization of AVIL and F-actin. Three representative images are shown.

Supplemental Figure 13.

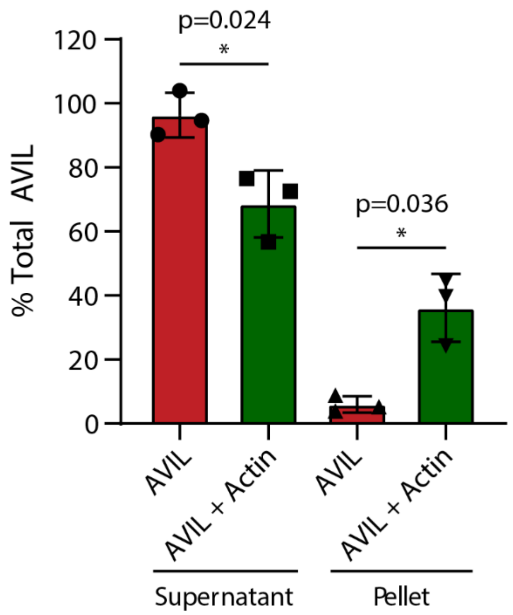

Supplementary Figure 13. Quantification of the results from actin binding assays. Graph representing quantification of experiment shown in actin binding assay in Figure 5C. Mean ± S.D are shown. * $p < 0.05$ (two-sided Student's t-test).

Supplemental Figure 14.

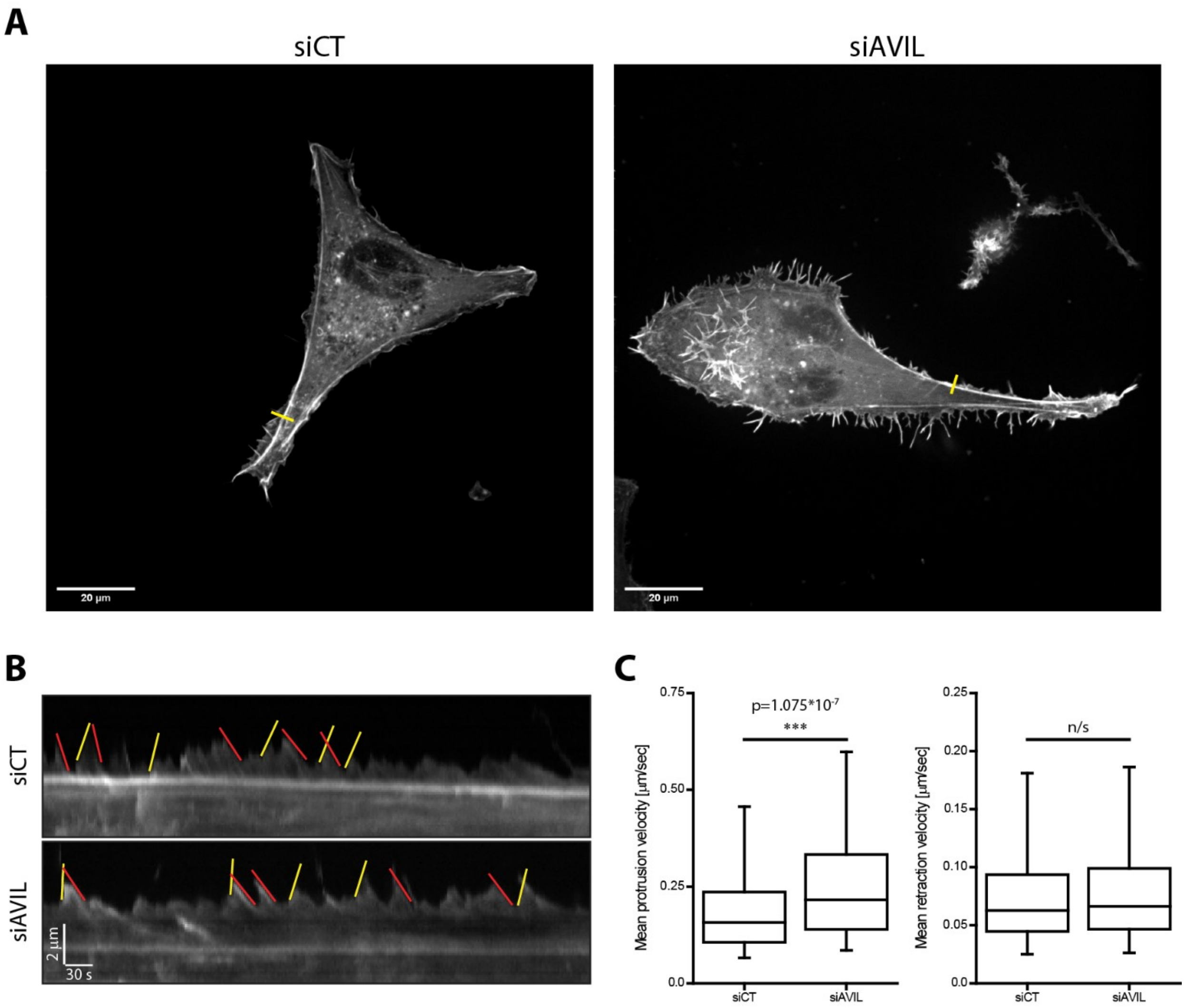

Supplementary Figure 14. Live cell imaging of A172 cells expressing mCherry-Lifeact. (A) Representative images of the cells at the first timepoint, 24hrs after transfection with either siGL2 (left) or siAVIL (right). White line indicates representative position used for further analysis. (B) Representative kymographs obtained from live-cell imaging as shown in panel A. Yellow lines represent the forward protrusions at the cell edges, while the red lines represent the retraction. (C) Quantification of the rates of membrane protrusions (left) and membrane retraction (right). n >250 events quantified per condition across >4 cells each condition (box, 25-75 percentile; whisker, 5-95 percentile; bar in middle, median) (two-sided Student's t-test). *** $p < 0.001$.

Supplemental Figure 15.

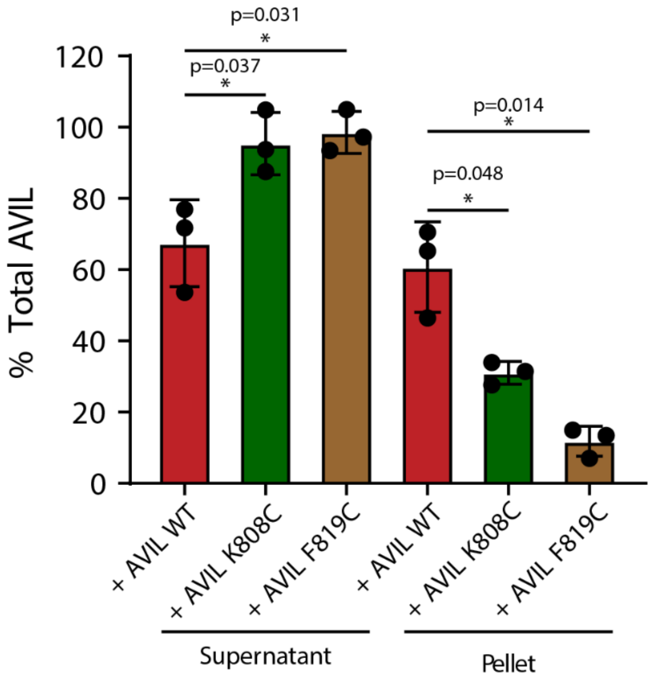

Supplementary Figure 15. Quantification of the results from actin binding assays. Graph representing quantification of the experiment shown in actin binding assay in Figure 5H. Mean ± S.D are shown (two-sided Student's t-test). * $p < 0.05$.

Supplemental Figure 16.

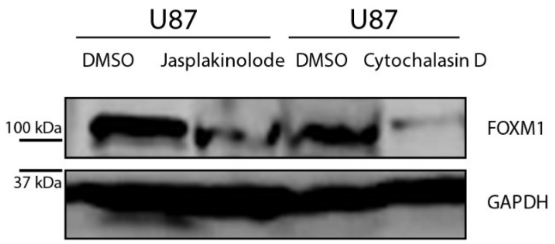

Supplementary Figure 16. FOXM1 protein is affected by F-actin dynamic inhibitors. F-actin polymerization inhibitor Cytochalasin D (1 µg/ml), and depolymerization inhibitor Jasplakinolide (150 nM) were used to treat U87 cells for 12 hrs. Western blot was performed with FOXM1 antibody to detect FOXM1 level change compared to solvent treatment controls (DMSO).

Supplemental Figure 17.

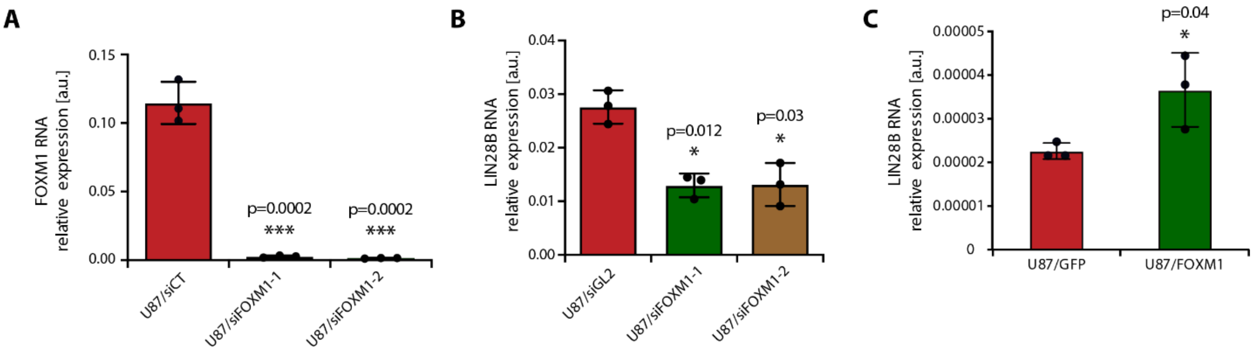

Supplementary Figure 17. LIN28B is downstream target of FOXM1. (A) FOXM1 was silenced by siRNAs. (B) LIN28B was suppressed upon FOXM1 silencing. (C) LIN28B was induced upon FOXM1 overexpression. *FOXM1*, and *LIN28B* levels were measured by qRT-PCR, and normalized to that of *GAPDH*. P value was calculated by standard two-tailed t-test. * p<0.05, ** p<0.01, *** p<0.001. Data are presented as mean values +/- SD.

Supplemental Figure 18.

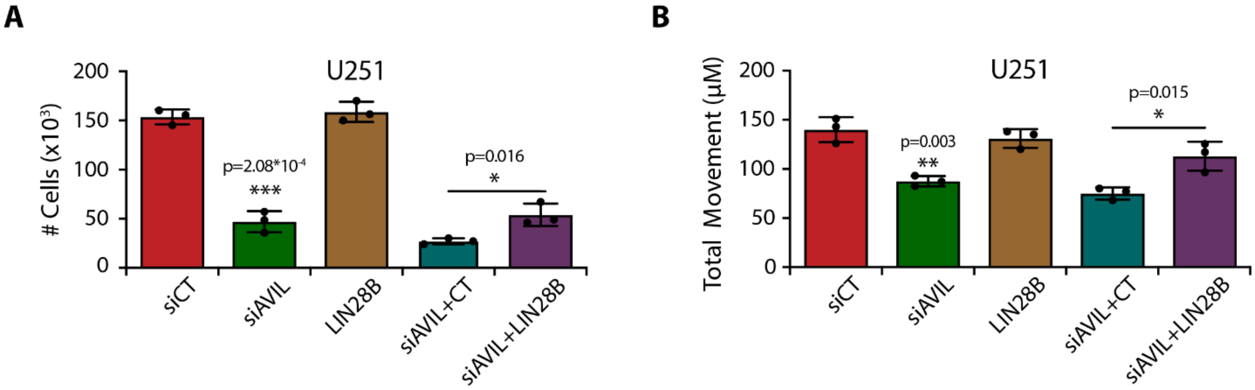

Supplementary Figure 18. LIN28B rescues at least some phenotype caused by silencing AVIL in U251. U251 cells transfected with siAVIL or siCT were further transfected with *LIN28B* expression vector, or control plasmid (CT). Cell proliferation was measured by cell counting (A) (two-sided Student's t test). Cell motility was measured by wound-healing assay (B) (two-sided Student's t test). Data are presented as mean values +/- SD. * $p<0.05$, ** $p<0.01$, *** $p<0.001$

Supplemental Figure 19.

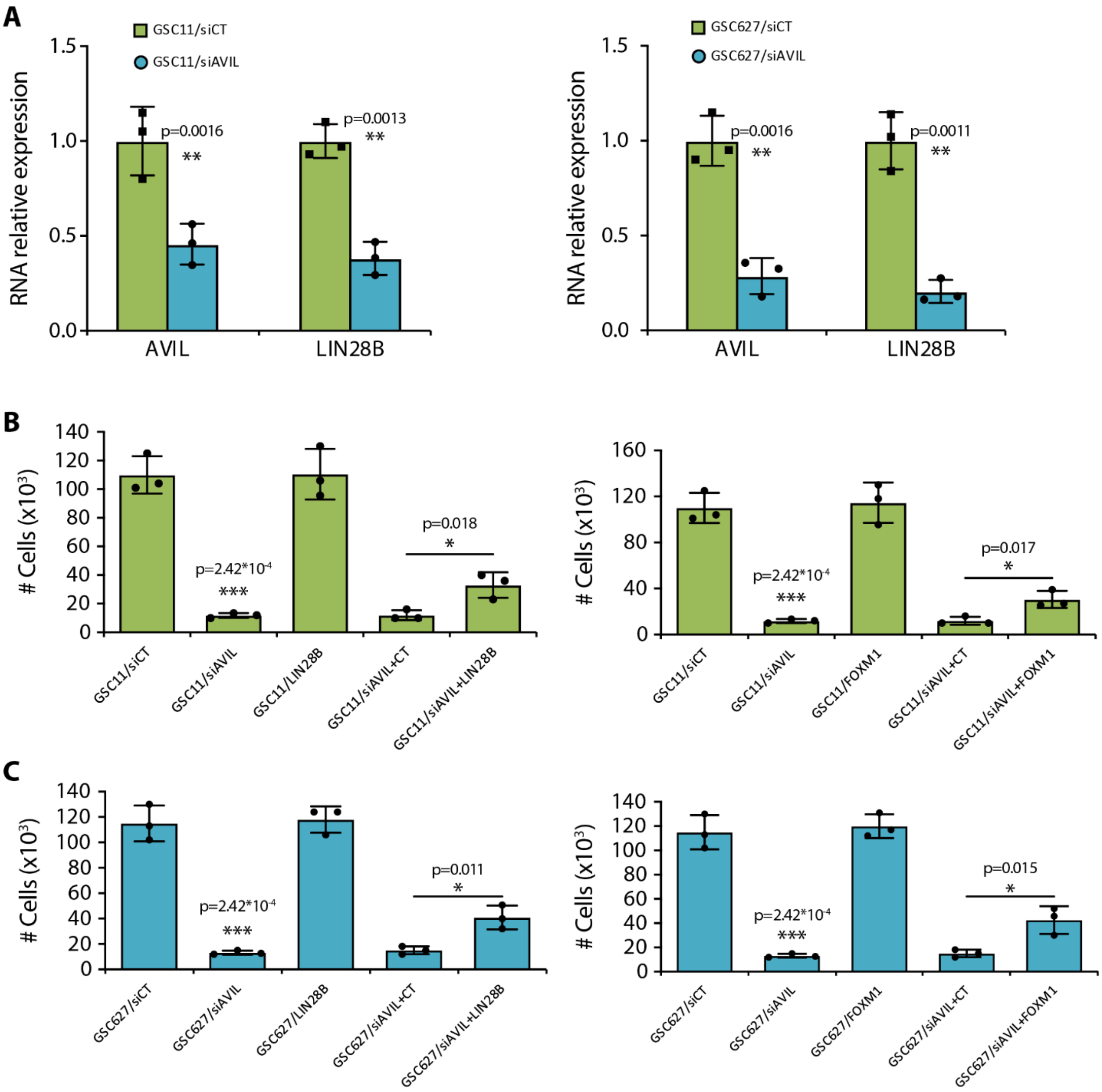

Supplementary Figure 19. LIN28B is downstream of AVIL in GSC cells. (A) qRT-PCR measuring *AVIL,* and LIN28B levels in GSC11 (left) and GSC627 (right) transfected with siAVIL, or control shCT. Various transcripts were normalized against that of *GAPDH*, and further normalized to the level in shCT group. (B and C) LIN28B and FOXM1 can partially rescue reduced proliferation caused by silencing AVIL in GSC11 (B) and GSC627 (C) cells. GSC cells transfected with siAVIL or siCT were further transfected with *LIN28B* expression vector (left), FOXM1 expression vector (right), or control plasmid (CT). Cell proliferation was measured by cell counting P value was calculated by standard two-tailed t-test. * p<0.05, ** p<0.01, *** p<0.001. Data are presented as mean values +/- SD.

Supplemental Figure 20.

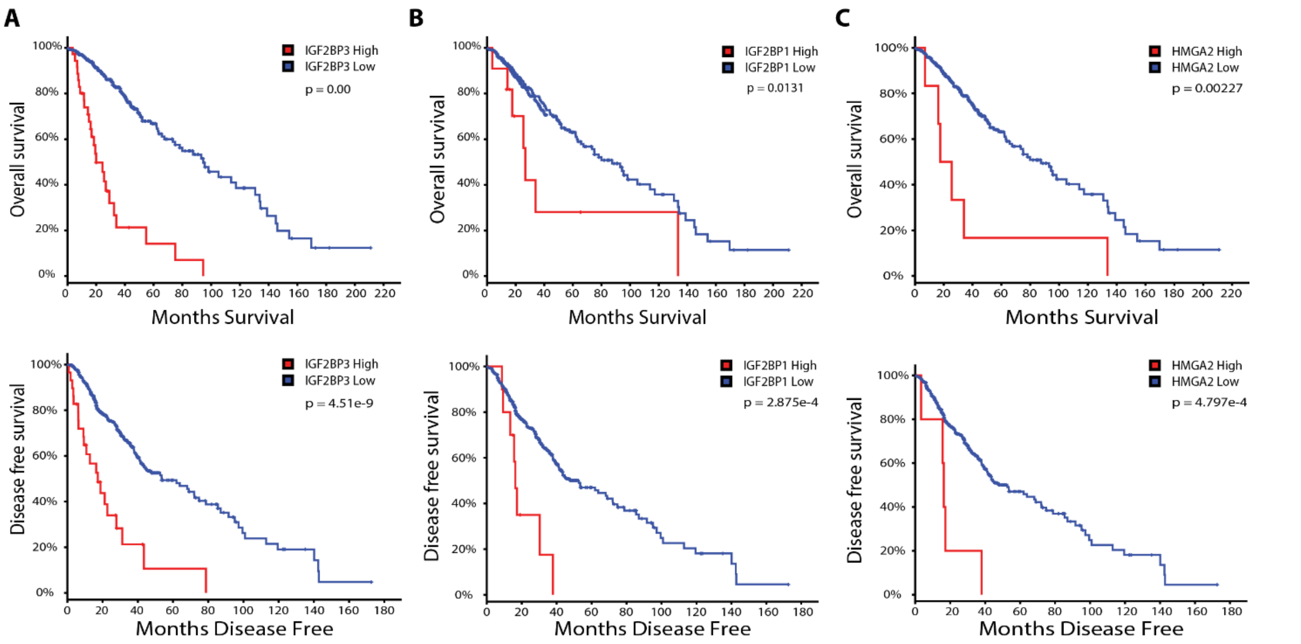

Supplementary Figure 20. Clinical correlation between the expression of let-7 targets with patient survival. (A-C) two-class models stratified by *IGF2BP3* (A), *IGFBP1* (B), and *HMGA2* (C) *and* expression in TCGA lower grade glioma cases. Patients with higher expression of any of the three genes have worse survival (two-sided log-rank test).

Supplemental Figure 21.

Fig. 1C

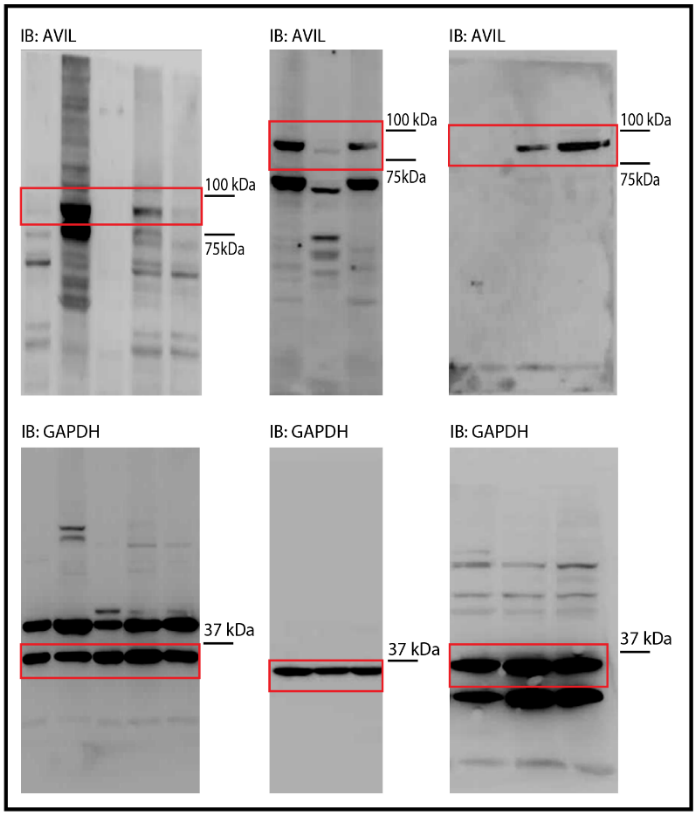

Fig. 1H

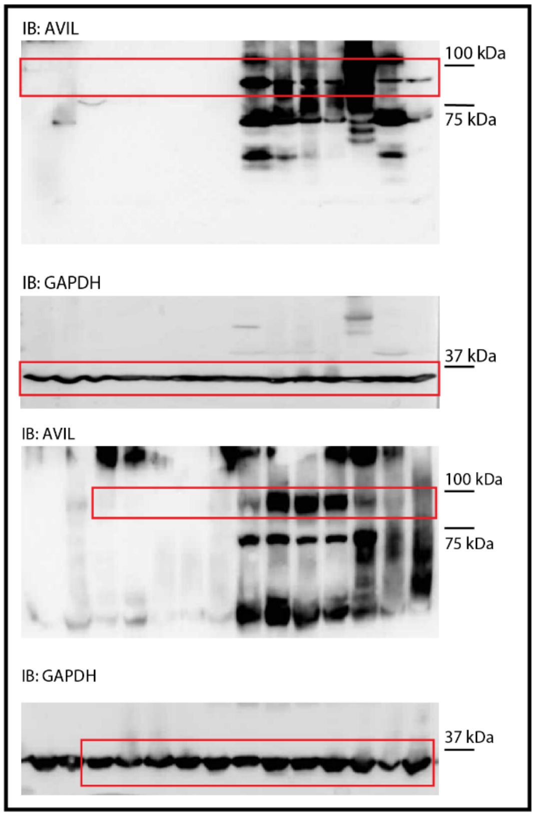

Fig. 3B

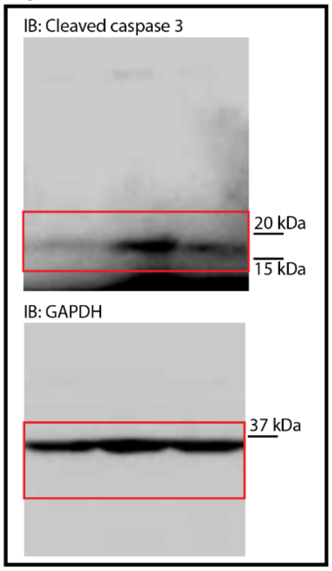

Fig. 4B

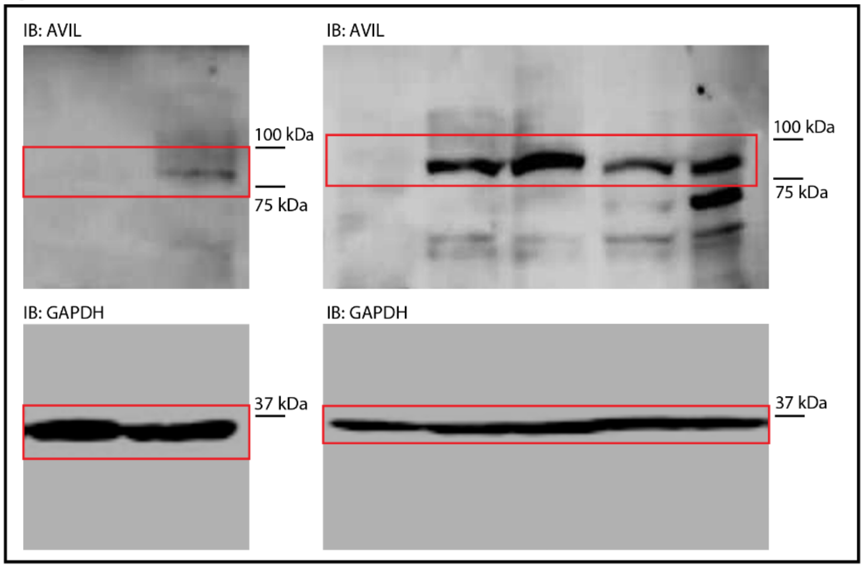

Supplementary Figure 21. Full scan of Western blots used in the figures. (continued on the next page)

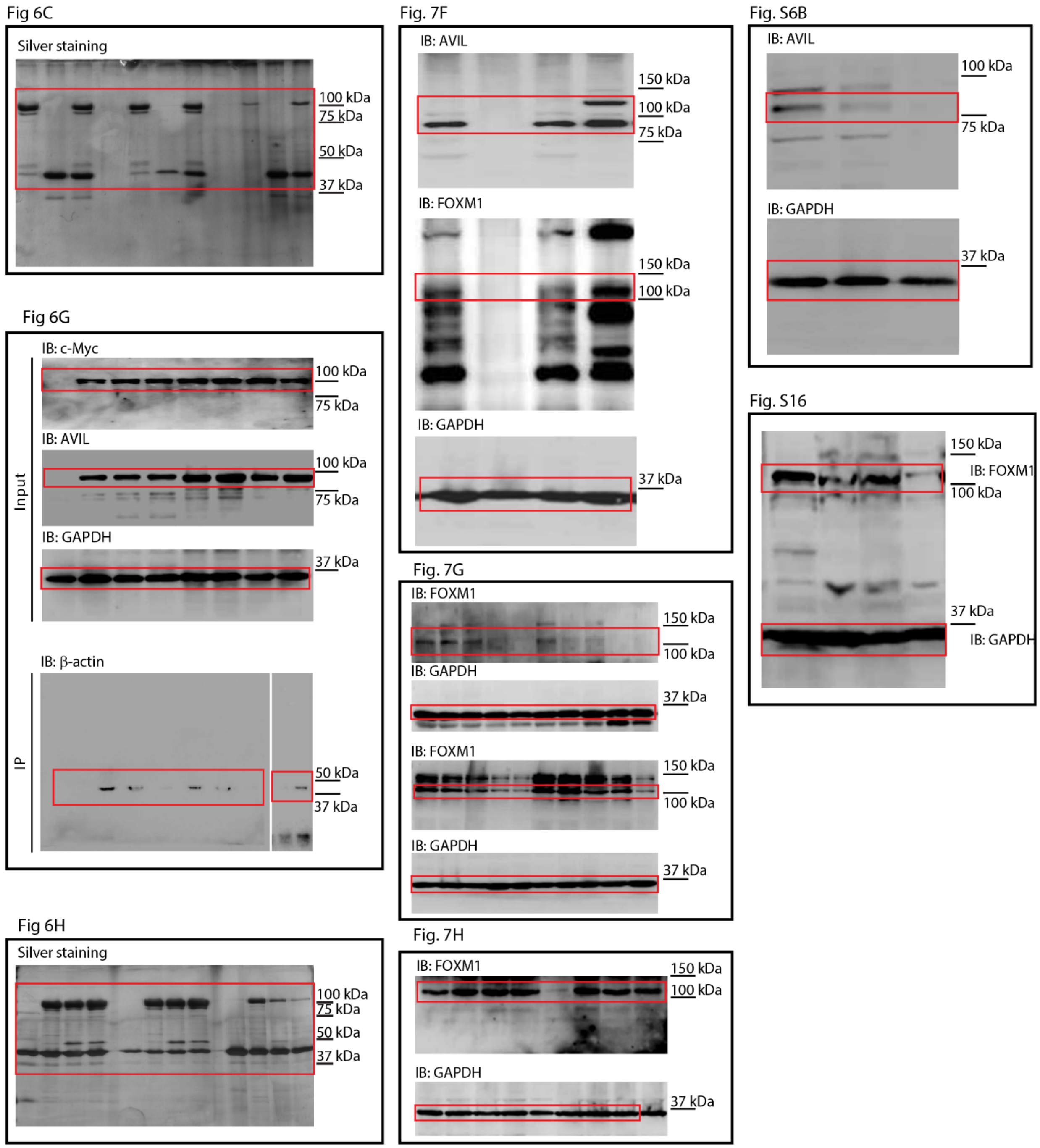

Supplementary Figure 21. Full scan of Western blots used in the figures.

SUPPLEMENTARY TABLE

Supplementary Table1, Primers used in the study

**Gene name**	**Forward primer**	**Reverse primer**
GAPDH	CTTTGGTATCGTGGAAGGACTC	GTAGAGGCAGGGATGATGTTCT
AVIL	GGCTGCTGTGCAGGTTCGA	AGGAGGCAAAGGCTGGAACT
FOXM1	GGAGGAAATGCCACACTTAGCG	TAGGACTTCTTGGGTCTTGGGGTG
LIN28B	CCTGTTTAGGAAGTGAAAGAAGAC	CACTTCTTTGGCTGAGGAGGTAG
CD133	GGACCCATTGGCATTCTC	CAGGACACAGCATAGAATAATC
SOX2	ACCTACAGCATGTCCTACTC	CATGCTGTTTCTTACTCTCCTC
GFAP	CAAAAGCACCAAAGACGGGG	GAGGCTCACTCCCTGTCAAG
Claudin-11	CTGGTGGACATCCTCATCCT	CCAGCAGAATGAGCAAAACA
TUJ1	GGCCAAGGGTCACTACACG	GCAGTCGCAGTTTTCACACTC
P53	CCTCAGCATCTTATCCGAGTGG	TGGATGGTGGTACAGTCAGAGC
RB	CAGAAGGTCTGCCAACACCAAC	TTGAGCACACGGTCGCTGTTAC
EGFR	AACACCCTGGTCTGGAAGTACG	TCGTTGGACAGCCTTCAAGACC
U6	CTCGCTTCGGCAGCACA	GAGACTGCGGATGTATAGAACTTGA
hsa-let-7a-5p	TGAGGTAGTAGGTTGTATAGTT	GAGACTGCGGATGTATAGAACTTGA
hsa-let-7b-5p	TGAGGTAGTAGGTTGTGTGGTT	GAGACTGCGGATGTATAGAACTTGA
hsa-let-7c-5p	TGAGGTAGTAGGTTGTATGGTT	GAGACTGCGGATGTATAGAACTTGA
hsa-let-7d-5p	AGAGGTAGTAGGTTGCATAGTT	GAGACTGCGGATGTATAGAACTTGA
hsa-let-7e-5p	TGAGGTAGGAGGTTGTATAGTT	GAGACTGCGGATGTATAGAACTTGA
hsa-let-7f-5p	TGAGGTAGTAGATTGTATAGTT	GAGACTGCGGATGTATAGAACTTGA
hsa-let-7g-5p	TGAGGTAGTAGTTTGTACAGT	GAGACTGCGGATGTATAGAAC

	T	TTGA
hsa-let-7i-5p	TGAGGTAGTAGTTTGTGCTGT T	GAGACTGCGGATGTATAGAAC TTGA
hsa-miR-98-5p	TGAGGTAGTAAGTTGTATTGT T	GAGACTGCGGATGTATAGAAC TTGA
