# Supplementary material for: A cytoskeleton regulator AVIL drives tumorigenesis in glioblastoma
Source: Nat Commun. 2020 Jul 10;11:3457. doi: 10.1038/s41467-020-17279-1 (PMC7351761; doi:10.1038/s41467-020-17279-1)
Supplement: Supplementary file 2 — Description of Additional Supplementary Files [file 41467_2020_17279_MOESM2_ESM.pdf]

Description of Additional Supplementary Files

File Name: Supplementary Movie 1
Description: **Live cell imaging of A172 cells.** A172 cells were transfected with siGL2 or siAVIL. 24 hours after siRNA transfection, images were collected every 20 minutes over the period of 24 hours. Cell movement was tracked semi-automatically based on DNA staining by TrackMate plugin for ImageJ.

File Name: Supplementary Movie 2
Description: **Live cell imaging of GSC11 cells.** GSC11 cells were transfected with siGL2 or siAVIL, then moved to MatriGel two hours later. Six hours after siRNA transfection, images were collected every 20 minutes over the period of 24 hours. Cell movement was tracked semi-automatically based on DNA staining by TrackMate plugin for ImageJ.
