# Supplementary material for: A cytoskeleton regulator AVIL drives tumorigenesis in glioblastoma
Source: Nat Commun. 2020 Jul 10;11:3457. doi: 10.1038/s41467-020-17279-1 (PMC7351761; doi:10.1038/s41467-020-17279-1)
Supplement: Supplementary file 5 — Reporting Summary [file 41467_2020_17279_MOESM5_ESM.pdf]

nature research

Corresponding author(s): Hui Li

Last updated by author(s): 2020-6-9

Reporting Summary

Nature Research wishes to improve the reproducibility of the work that we publish. This form provides structure for consistency and transparency in reporting. For further information on Nature Research policies, see our Editorial Policies and the Editorial Policy Checklist.

Statistics

For all statistical analyses, confirm that the following items are present in the figure legend, table legend, main text, or Methods section.

n/a	Confirmed	
☐	☒	The exact sample size (*n*) for each experimental group/condition, given as a discrete number and unit of measurement
☐	☒	A statement on whether measurements were taken from distinct samples or whether the same sample was measured repeatedly
☐	☒	The statistical test(s) used AND whether they are one- or two-sided<br>*Only common tests should be described solely by name; describe more complex techniques in the Methods section.*
☒	☐	A description of all covariates tested
☐	☒	A description of any assumptions or corrections, such as tests of normality and adjustment for multiple comparisons
☐	☒	A full description of the statistical parameters including central tendency (e.g. means) or other basic estimates (e.g. regression coefficient) AND variation (e.g. standard deviation) or associated estimates of uncertainty (e.g. confidence intervals)
☐	☒	For null hypothesis testing, the test statistic (e.g. *F*, *t*, *r*) with confidence intervals, effect sizes, degrees of freedom and *P* value noted<br>*Give P values as exact values whenever suitable.*
☒	☐	For Bayesian analysis, information on the choice of priors and Markov chain Monte Carlo settings
☒	☐	For hierarchical and complex designs, identification of the appropriate level for tests and full reporting of outcomes
☒	☐	Estimates of effect sizes (e.g. Cohen's *d*, Pearson's *r*), indicating how they were calculated

Our web collection on statistics for biologists contains articles on many of the points above.

Software and code

Policy information about availability of computer code

Data collection	qRT-PCR: StepOne Plus; MATLAB R2016a; ImageJ 1.52a
Data analysis	GraphPad Prism 8.0.1; Microsoft Exel 16.131; Photoshop 2015.1.2; ImageJ 1.52a; MATLAB R2016a

For manuscripts utilizing custom algorithms or software that are central to the research but not yet described in published literature, software must be made available to editors and reviewers. We strongly encourage code deposition in a community repository (e.g. GitHub). See the Nature Research guidelines for submitting code & software for further information.

Data

Policy information about availability of data

All manuscripts must include a data availability statement. This statement should provide the following information, where applicable:

- Accession codes, unique identifiers, or web links for publicly available datasets
- A list of figures that have associated raw data
- A description of any restrictions on data availability

The Raw and processed RNA-Sequencing data from this study have been submitted to the NCBI Gene Expression Omnibus (GEO; http://www.ncbi.nlm.nih.gov/geo/) under accession number GSE64032. Microarray data for this study has also been deposited onto the GEO database, GSE95164. The other data that support the findings of this study are available from the corresponding author upon reasonable request.

Field-specific reporting

Please select the one below that is the best fit for your research. If you are not sure, read the appropriate sections before making your selection.

☒ Life sciences ☐ Behavioural & social sciences ☐ Ecological, evolutionary & environmental sciences

For a reference copy of the document with all sections, see nature.com/documents/nr-reporting-summary-flat.pdf

Life sciences study design

All studies must disclose on these points even when the disclosure is negative.

Sample size	Sample size for each experiment is indicated in the legend or methods. No statistical tests were used to pre-determine sample size. Sample size was chosen based on previous experiments and comparable studies in literature, which is most optimal to generate statistically significant results.
Data exclusions	No data was excluded for analysis.
Replication	Each animal experiment presented in the paper was repeated in multiple animals (>= 6 per experiment) , and all results in the paper are drawn from the analysis of multiple animals. For other experiments, all attempts at replication were successful. The number of replications was stated in the figure legends of Methods.
Randomization	For animal experiments, animals were assigned randomly to experimental and control groups. Cells were all plated at the same time and wells were randomly selected for different treatment with stimuli or inhibitors.
Blinding	Blinding was not applicable as it was not a randomized clinical trial.

Reporting for specific materials, systems and methods

We require information from authors about some types of materials, experimental systems and methods used in many studies. Here, indicate whether each material, system or method listed is relevant to your study. If you are not sure if a list item applies to your research, read the appropriate section before selecting a response.

Materials & experimental systems

n/a	Involved in the study	
☐	☒	Antibodies
☐	☒	Eukaryotic cell lines
☒	☐	Palaeontology and archaeology
☐	☒	Animals and other organisms
☒	☐	Human research participants
☒	☐	Clinical data
☒	☐	Dual use research of concern

Methods

n/a	Involved in the study	
☒	☐	ChIP-seq
☒	☐	Flow cytometry
☐	☒	MRI-based neuroimaging

Antibodies

Antibodies used	rabbit anti-MARS (Sigma HPA004125), rabbit anti-AVIL (Abcam ab72210), rabbit anti-PARP (Cell Signaling 9542), rabbit anti-Cleaved Caspase-3 (Cell Signaling 9664), and mouse anti-GAPDH (Ambion Am4300), rabbit anti- Ki67 (Abcam, ab16667)
Validation	Antibody validation was deferred to the manufacturers and was supported by multiple publications.

Eukaryotic cell lines

Policy information about cell lines

Cell line source(s)	U87, A712, T98G (ATCC); NSC (Millipore); GSC/GIC cells (gifts from Dr. Krishna Bhat, MD Anderson); Immortalized human astrocytes (gift from Dr. Russ Pieper, University of California San Francisco).
Authentication	None of the cell lines have been authenticated.
Mycoplasma contamination	Cell lines were not tested for mycoplasma contamination but no indication of contamination was observed.
Commonly misidentified lines (See ICLAC register)	No commonly misidentified cell lines were used.

Animals and other organisms

Policy information about studies involving animals; ARRIVE guidelines recommended for reporting animal research

Laboratory animals	Immunocompromised SCID/NCr BALB/c adult male mice (6-8 weeks old) were used.
Wild animals	No wild animals were used in this study.
Field-collected samples	No field-collected samples were used in this study.
Ethics oversight	The mouse work was performed under the study protocol 4234 as approved by the University of Virginia Institutional Animal Care and Use Committee.

Note that full information on the approval of the study protocol must also be provided in the manuscript.

Magnetic resonance imaging

Experimental design

Design type	Measurement of tumor growth in GBM-tumor bearing mice.
Design specifications	In each session of MRI imaging, coronal images of the brain tumor were acquired in each mouse. It took 30 mins per mouse.
Behavioral performance measures	Mice were sedated for the MRI imaging of the GBM tumor. The heart rate was monitored during the imaging to ensure the heart rate remained constant.

Acquisition

Imaging type(s)	Structural
Field strength	7Tesla
Sequence & imaging parameters	T1-weighted serial coronal images of each brain were acquired at 1 mm intervals with a 5 x 5 mm field, and a 256 x 256 pixel resolution.
Area of acquisition	Whole brain was scanned.

Diffusion MRI ☐ Used ☒ Not used

Preprocessing

Preprocessing software	ImageJ version 1.52a was used for measuring tumor volumes. All brain sections for each mouse was imported as an image sequence.
Normalization	All images from all mice were adjusted for equal brightness and contrast.
Normalization template	No specific template was used.
Noise and artifact removal	A luminosity histogram was first generated for a selected area of the left cerebrum that was grossly tumor-free. This served as an internal control for background.
Volume censoring	No volume censoring was done.

Statistical modeling & inference

Model type and settings	No statistical modeling was done with these images.
Effect(s) tested	*Define precise effect in terms of the task or stimulus conditions instead of psychological concepts and indicate whether ANOVA or factorial designs were used.*

Specify type of analysis: ☐ Whole brain ☐ ROI-based ☐ Both

Statistic type for inference (See Eklund et al. 2016)	*Specify voxel-wise or cluster-wise and report all relevant parameters for cluster-wise methods.*
Correction	*Describe the type of correction and how it is obtained for multiple comparisons (e.g. FWE, FDR, permutation or Monte Carlo).*

Models & analysis

n/a	Involved in the study
☒	☐ Functional and/or effective connectivity
☒	☐ Graph analysis
☒	☐ Multivariate modeling or predictive analysis
